# Temporal Dynamics of Host Molecular Responses Differentiate Symptomatic and Asymptomatic Influenza A Infection

**DOI:** 10.1371/journal.pgen.1002234

**Published:** 2011-08-25

**Authors:** Yongsheng Huang, Aimee K. Zaas, Arvind Rao, Nicolas Dobigeon, Peter J. Woolf, Timothy Veldman, N. Christine Øien, Micah T. McClain, Jay B. Varkey, Bradley Nicholson, Lawrence Carin, Stephen Kingsmore, Christopher W. Woods, Geoffrey S. Ginsburg, Alfred O. Hero

**Affiliations:** 1 Center for Computational Biology and Bioinformatics, University of Michigan, Ann Arbor, Michigan, United States of America; 2 Department of Statistics, University of Michigan, Ann Arbor, Michigan, United States of America; 3 Institute for Genome Sciences and Policy, Duke University, Durham, North Carolina, United States of America; 4 Department of Medicine, Duke University, Durham, North Carolina, United States of America; 5 Lane Center for Computational Biology, Carnegie Mellon University, Pittsburgh, Pennsylvania, United States of America; 6 IRIT/INP-ENSEEIHT, University of Toulouse, Toulouse, France; 7 Department of Biomedical Engineering, University of Michigan, Ann Arbor, Michigan, United States of America; 8 Department of Chemical Engineering, University of Michigan, Ann Arbor, Michigan, United States of America; 9 Department of Medicine, School of Medicine, Emory University, Atlanta, Georgia, United States of America; 10 Department of Electrical and Computer Engineering, Duke University, Durham, North Carolina, United States of America; 11 Center for Pediatric Genomic Medicine, Children's Mercy Hospital, Kansas City, Missouri, United States of America; 12 Department of Electrical Engineering and Computer Science, University of Michigan, Ann Arbor, Michigan, United States of America; University of California San Diego and The Scripps Research Institute, United States of America

## Abstract

Exposure to influenza viruses is necessary, but not sufficient, for healthy human hosts to develop symptomatic illness. The host response is an important determinant of disease progression. In order to delineate host molecular responses that differentiate symptomatic and asymptomatic Influenza A infection, we inoculated 17 healthy adults with live influenza (H3N2/Wisconsin) and examined changes in host peripheral blood gene expression at 16 timepoints over 132 hours. Here we present distinct transcriptional dynamics of host responses unique to asymptomatic and symptomatic infections. We show that symptomatic hosts invoke, simultaneously, multiple pattern recognition receptors-mediated antiviral and inflammatory responses that may relate to virus-induced oxidative stress. In contrast, asymptomatic subjects tightly regulate these responses and exhibit elevated expression of genes that function in antioxidant responses and cell-mediated responses. We reveal an *ab initio* molecular signature that strongly correlates to symptomatic clinical disease and biomarkers whose expression patterns best discriminate early from late phases of infection. Our results establish a temporal pattern of host molecular responses that differentiates symptomatic from asymptomatic infections and reveals an asymptomatic host-unique non-passive response signature, suggesting novel putative molecular targets for both prognostic assessment and ameliorative therapeutic intervention in seasonal and pandemic influenza.

## Author Summary

The transcriptional responses of human hosts towards influenza viral pathogens are important for understanding virus-mediated immunopathology. Despite great advances gained through studies using model organisms, the complete temporal host transcriptional responses in a natural human system are poorly understood. In a human challenge study using live influenza (H3N2/Wisconsin) viruses, we conducted a clinically uninformed (unsupervised) factor analysis on gene expression profiles and established an *ab initio* molecular signature that strongly correlates to symptomatic clinical disease. This is followed by the identification of 42 biomarkers whose expression patterns best differentiate early from late phases of infection. In parallel, a clinically informed (supervised) analysis revealed over-stimulation of multiple viral sensing pathways in symptomatic hosts and linked their temporal trajectory with development of diverse clinical signs and symptoms. The resultant inflammatory cytokine profiles were shown to contribute to the pathogenesis because their significant increase preceded disease manifestation by 36 hours. In subclinical asymptomatic hosts, we discovered strong transcriptional regulation of genes involved in inflammasome activation, genes encoding virus interacting proteins, and evidence of active anti-oxidant and cell-mediated innate immune response. Taken together, our findings offer insights into influenza virus-induced pathogenesis and provide a valuable tool for disease monitoring and management in natural environments.

## Introduction

Influenza viruses are highly infectious and can cause acute respiratory illness in human hosts. Infected hosts present a variety of clinical symptoms including fever, runny nose, sore throat, myalgias, and malaise with potentially more serious complications such as viral pneumonia [1]. Many hosts also withstand comparable level of viral insult with little or no overt symptoms, exhibiting a higher degree of tolerance [2], [3]. Clearly, these asymptomatic infected hosts are able to control and eradicate viral threats more effectively than those who become symptomatic. Given the dynamic nature of viral infection, it is now recognized that interactions between hosts and viruses play a crucial role in determining the presence and absence of symptoms [4]. This leads to an interesting question ― what are the principal factors associated with such divergent disease outcome?

In recent years, seminal studies on the sensing of pathogens by pattern-recognition receptors (PRRs) and their related signaling cascades have advanced our understanding of innate immunity [5]–[10]. Many elegant experimental analyses have further elucidated the mechanistic activation and modulation of host response to invading pathogens [11]–[16]. By design, however, host responses in these experimental conditions are often characterized for individual cells via cell culture; or they represent a snapshot of the immune response pertaining to a limited number of time points. The components of the host immune system are diverse and they interact in a complicated manner. Owing to both technical and ethical difficulties, it has not been practical to experimentally determine the full course of immune responses leading to severe symptoms in otherwise healthy human hosts. Thus the time sequence and orchestration of host response events remain to be fully understood.

The peripheral blood contains key elements of the immune system and the circulating immune cells recruited by the host in response to viral infection and virus-induced tissue damage provides a global view of the host immune response. Thus, we hypothesized that it can be used to monitor the temporal dynamics of host-virus interactions. Analyzing whole-genome gene expression profiles from healthy human subjects challenged with influenza H3N2/Wisconsin, we studied the full temporal spectrum of virus-mediated disease dynamics. Going beyond the peak symptom time analysis reported in Zaas et al. [17], this report offers an hour-by-hour detailed view of host immune response as a continuum, spanning the time from exposure to peak symptom manifestation. Utilizing biological and mathematical models, we highlight key immune response events representing potential factors that determine the pathogenicity of influenza viral infection. We further present a statistical risk-stratification model for estimating current disease state with potential forward risk assessment capability. These results are concordant with findings reported by Zaas et al. that was limited to peak symptom time analysis.

## Results

### Outline of overall analysis strategy

A cohort of 17 healthy human volunteers (Table S1) received intranasal inoculation of influenza H3N2/Wisconsin and 9 of these subjects developed mild to severe symptoms based on standardized symptom scoring [18]. Gene expression profiles were measured on whole peripheral blood drawn from all subjects at an interval of ∼8 hours post inoculation (hpi) through 108 hpi. A total of 267 gene expression profiles were obtained for all subjects at 16 time points including baseline (−24 hpi). As outlined in Figure S16, our analysis of the data consists of two parallel components: 1) clinically uninformed (unsupervised) factor analysis using Bayesian Linear Unmixing (BLU) [19]; 2) clinically informed (supervised) pathway analysis using EDGE [20] and self organizing maps (SOM) [21] that leverages clinical and temporal covariates for increased statistical power. The former establishes the existence of an *ab initio* molecular signature that strongly correlates to symptomatic clinical disease. The later further reveals important host factors that delineate time courses of designated symptomatic (Sx) and asymptomatic (Asx) subjects.

### A genomic signature discriminates between early and late stages of disease

**Figure 1:**
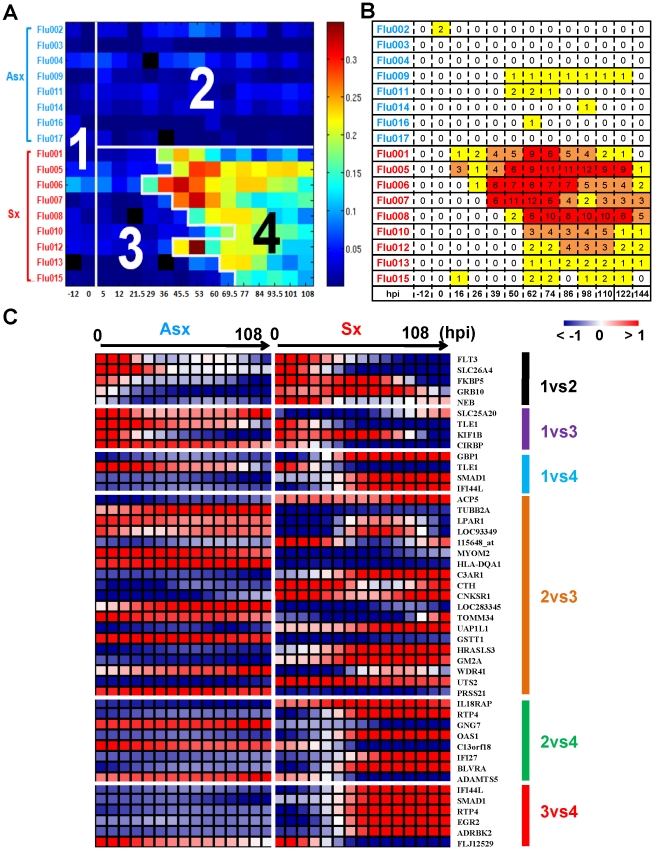
The BLU genetic signature correlates strongly with disease severity and yields early- and late-stage risk stratification model. (A) The scores of the top ranked factor detected by the unsupervised BLU factor analysis method. Each microarray sample is represented by one square cell of the image and ordered by phenotype and subject (row-wise) and increasing time (column-wise). Color palette is coded according to the enrichment factor score determined by BLU. The higher the score, the warmer (red) color representation of the sample. The numbers (1 to 4) are the disease state (class) designation determined by BLU (3 and 4) and inoculation time (1 and 2). The boundary between 3 and 4 occurs at samples that are labeled (To) denoting the critical transition point (onset time) of Sx subject transcriptome profiles. The grey color (absolute 0 loading) corresponds to samples that were not assayed. (B) Clinical symptom chart of corresponding subjects (rows) and times (columns) that are ordered in the same manner as A. (C) Heatmap of groups of genes that are most highly associated with differences between pairs of classes according to a logistic regression model. For purposes of visualization, the heatmaps show gene expression profiles that are averaged over Asx (left) and Sx (right) phenotypes and are smoothed over time using the same cubic spline fitting method as used for heatmaps shown in Figure 2B.

Symptomatic infection exhibits a distinct time evolving molecular signature. This signature is sufficiently strong that a clinically uninformed factor analysis method is able to pick it up without using any clinical phenotype information such as disease outcome, subject or time labels. For this analysis we used the BLU factor analysis method described in the Methods section. Figure 1A shows a heatmap of the linear combination (BLU factor score) of genes in this signature, where for visualization we have arranged the samples in a matrix whose rows and columns are organized according to clinical phenotype of the subject and sample time. The image of the BLU factor score shown in Figure 1A bears striking resemblance to the standardized clinical symptom observation matrix in Figure 1B.

The BLU factor score signature is sufficiently strong that application of a threshold to the post-inoculation part of the heatmap in Figure 1A perfectly divides the subjects into asymptomatic subjects (Class 2) and symptomatic subjects before onset (Class3) and after onset (Class 4) of acute infection. The selection of the threshold was based on the pre-inoculation samples (Class 1) and is described in the Methods section. Then, using logistic regression [22] as an association measure between class label and gene expression, we extracted sets of genes that are most associated with differences between pairs of classes (Table S4). When the expression profiles of these genes are plotted as heatmaps (Figure 1C) the contrasts in gene expression are striking. For example, the type-I interferon antiviral response related genes IFI44L, IFI27, GBP1, RTP4, and OAS1 are among the most associated with differentiating acute infection (class 4) from the other 3 classes. As another example, note the contrast between complement component 3a receptor (C3AR1) between Classes 2 and 3, exhibiting a marked change after inoculation in symptomatic subjects. These genes are well known for their critical function in host immunity [6], [23], [24]. This demonstrates both the strength of the genomic signature of acute infection and the utility of BLU factor analysis for *ab initio* discovery of this signature.

### Identification of eight distinct virus-mediated gene expression dynamics

**Figure 2:**
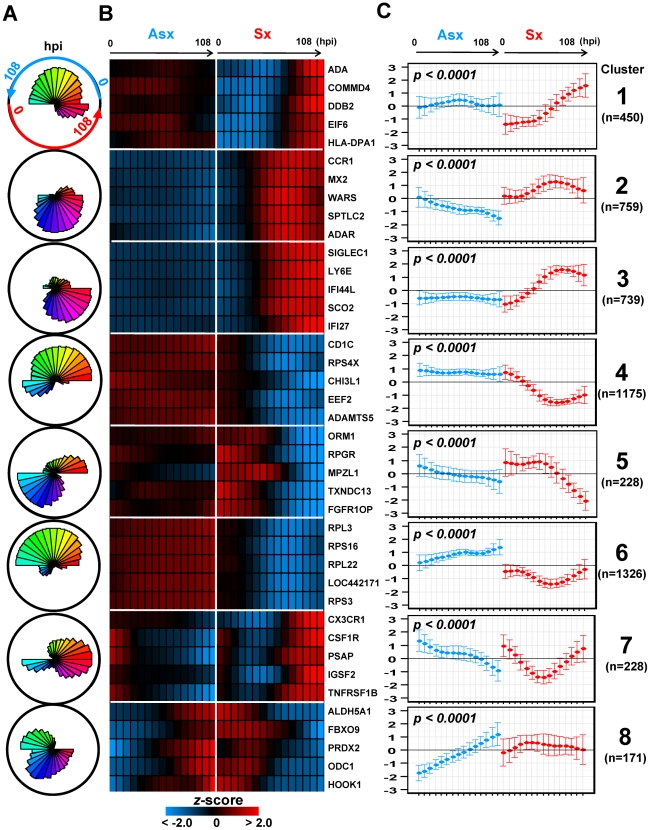
Self-organizing map clusters show distinct transcriptional dynamics in Influenza H3N2/Wisconsin virus challenge study. (A) Polar plots of the 8 SOM clusters and their associated gene expression patterns. Each segment plot represents the prototype of a cluster. Individual time points are scaled and ordered in sequence and phenotype around the circle. Specifically, the temporal expression of Asx resides on the top portion of the circle while Sx expression occupies the bottom half. Each phenotype's expression values are placed in time sequence, with time increasing in the counterclock-wise direction, inside its own half circle. The degrees of angle are equally divided among segments within the circular plot. The different lengths of radii of the segments represent the deviation of a time point from the average expression level of the complete time course. (B) Heatmaps of EDGE-estimated temporal profiles of the top 5 genes from each SOM cluster (EDGE averages over Asx (left) and Sx subjects (right) and smoothes over time using a fitted cubic spline). Genes in each cluster are ordered in decreasing order of EDGE significance level (See Figure S18 for heatmaps of all significant genes found by EDGE). (C) Centroids of each SOM cluster show individual cluster average expression profile and corresponding 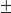
two standard deviations. The statistical significance of phenotype-specific trend of expression monotonicity can be found in Table S3. hpi: hours post inoculation.

When we add clinical and temporal information about the samples to the analysis we can identify clusters of genes whose temporal expression patterns differentiate immune response of clinically asymptomatic from clinically symptomatic subjects. Using EDGE with false discovery rate (FDR) significance level (*q*-value)<0.01, we selected 5,076 genes whose temporal expression profiles differed significantly between Asx and Sx phenotypes. Heatmaps of these 5,076 EDGE genes are shown in Figure S18. Next, these 5,076 gene expression profiles were grouped into clusters based on using SOM applied jointly to the Sx and Asx phenotypes. A total of eight clusters were identified and their associated centroids are shown in Figure 2A and 2C as polar and linear plots of expression over time. Heatmaps of gene expression are shown for the top 5 genes in each SOM cluster (Figure 2B). These eight clusters decompose temporal host response into eight distinct classes of differential expression dynamics, revealing divergent trends in asymptomatic and symptomatic responses over time. The contrasts in expression patterns between phenotypes are all statistically significant (*q*-value<0.01) (Figure 2C). Most clusters show significant monotonic increase or decrease in expression over time in Asx or Sx phenotypes (Table S3). For Sx subjects we define three stages of infection: early (0–12 hpi), middle (12–45 hpi), and late (>45 hpi).

Collectively, clusters 2, 3, 4, and 6 contain more than 78% of all significant genes and highlight the sharp contrasts in expression dynamics between phenotypes. Although the discussion below focuses on these four clusters, pathway enrichment analysis indicates that genes from all eight clusters are directly related to the activation and modulation of host immune and inflammatory responses (Table 1). Clusters 3 and 4 contain genes that are associated with equally strong Sx response but responded discordantly. Cluster 3 is denoted as (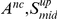) where superscripts *nc* and *up* stands for no change and upregulation, respectively. The subscript *mid* (middle stage) indicates the onset time of the change. Cluster 3 is characterized by strong activation, in Sx phenotype, of genes responsible for antiviral and inflammatory responses. Cluster 4, (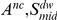), contains genes that are continuously down-regulated in the Sx phenotype in contrast to nearly no change in the Asx phenotype. On the other hand, genes in clusters 2 and 6 are associated with strong but discordant responses in both Asx and Sx individuals, indicating an active physiological response in Asx hosts. Cluster 2, (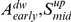), includes genes exhibiting sustained decrease unique to the Asx phenotype from early time onward. In Sx, the expression of cluster 2 genes increases to peak level at the middle of challenge (45–69 hpi), followed by a rescinding trend. Cluster 6, (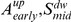), is populated by genes whose expression steadily increases in the asymptomatic phenotype over all time. In contrast, for the symptomatic subjects these genes exhibit a transient but significant decrease beginning at 29 hpi and return to baseline after 60 hpi.

**Table 1:** Canonical pathways and representative genes enriched in individual SOM clusters.

SOM Cluster	# of Genes	Pathway	Representative Genes
**1**	**450**	immune cell trafficking; antigen presentation	CD74, HLA-DMA, HLA-DPA1, HLA-DPB1, CCR5, CCL4, TBX21, IL10RA, CD244, ICAM2
**2**	**759**	inflmmation; chemotaxis of macrophage, neutrophils, and dendritic cells; antigen presentation, JAK-STAT signaling	SOCS1, SOCS3, NOD2, NLRP3, CASP5, IL1B, STAT3, ADM,C5, CCL2/7/8/11, CCR1, CCR4, CD14, CD59, CD163, CD209, CEACAM3, CXCL9, CXCL10, CXCL11, FAS, HLA-B, ICAM1, IL17RB, IL18R1, IL18RAP, LILRA2, LTBR, MX2, TGFB1, TLR1, TLR2, TLR4, TLR5, TLR8, TREM2, TRIM21, SERPINA1, CASP4, IFITM2
**3**	**739**	inflammatory response; dendritic cell and neutrophil activation; IFN-signaling	TLR7, MYD88, IRF7, IRF5, IRF9, TNF, JAK2, PSMB8, STAT1, DDX58, IFIH1, IL18, IL10, MX1, RSAD2, OAS1, SIGLEC1, NOD1, CASP1, PKR, TRIM22, LILRB1, ISG20, IFNAR1, IFI44, CD86, CD40, CD63, C1QA, IL10RB, TNFRSF14, TNFSF10, TNFSF12, BTK, RNASE2; C3AR1, CYBB, FASLG, APOL3, ANXA2, IFI35, IFIT1, IFIT3, IFITM1, IFITM3
**4**	**1175**	oxidative stress; ca+ induced T cell apoptosis; iCOS signaling	CCL5, RPS6KA5, ACTG1, CUL3, PRKC GENES, C-JUN, PIK3 family, MAP2K4, CD3E, CD247, CD40LG, CAMK4M, IL2RB, ITK, ITPR1, ITPR3, LAT, NFATC1, NFATC3, ICOS, FYN
**5**	**228**	antigen presentation; innate immune response	CD97, THBD, DDX17, IL1R2, ORM1, TREM1, AOC3, FOXO3, IL1R1, IL1RAP, AQP9, CA4, CAMK1D
**6**	**1326**	protein synthesis; oxidative stress; RNA trafficking; JAK-STAT signaling	SOCS2, SOCS5, SOD1, SOK1, RPL3, EIF3 FAMILY GENES, CCR7, RPS9, RPS14, RPL22, C1QBP, DDX21, DDX50, ICOS
**7**	**228**	natural killer cell signaling; cell apoptosis	SIGLEC7, ASC, SHC1, MAPK7, KIR2DL1, KIR2DS4, KIR3DL1, SERPINF1, RAC1, CD4, CX3CR1, HLA-G, TNFRSF1B, ITGB2, CTSD
**8**	**171**	cell morphology; cell signaling	EIF2AK1, LY96, BCL2L1, KRAS, PIM1, TGM2, RGS1, PKN2

### Host transcription signatures are highly correlated with disease dynamics

**Figure 3:**
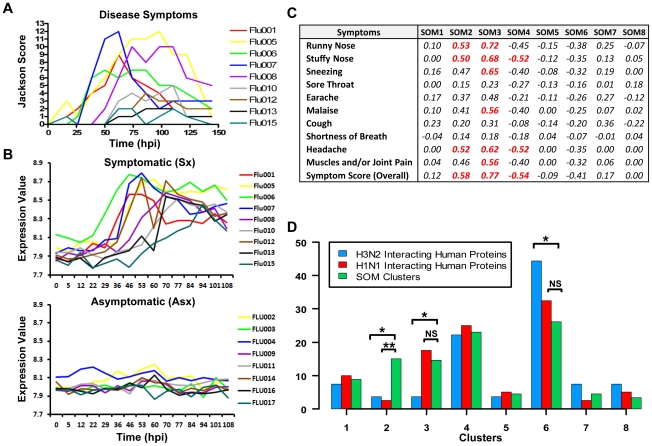
Strong correlation of molecular signature with disease severity. (A) Clinical symptom scores of symptomatic subjects with individuals represented by curves in different colors. (B) Cluster 3 gene expression of symptomatic subjects (top) and Asx subjects (bottom). (C) The correlation coefficients (total variance explained) between standardized symptom scores and SOM clusters. (D) Cluster-wise distribution of genes encoding proteins that are known to interact with H1N1/PR8 and H3N2/Udorn viruses. The proportion of genes encoding virus-human interaction proteins in a cluster is compared to the relative size of that cluster for determining significance of distributional differences (Fisher's exact test; *<0.05, **<0.005).

The eight clusters represent molecular signatures of unique and contrasting temporal dynamics. We evaluated whether these signatures are related to symptom development by correlating the expression of these signatures against standardized clinical symptom scores [17], [18]. Both positive and negative correlations were observed (Figure 3C). In particular, cluster 3 (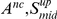) showed the strongest positive correlation with symptom scores (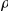 = 0.77) followed by cluster 2 (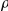 = 0.58). The temporal expression pattern of cluster 3 genes closely resembled the disease progression trajectory of each individual Sx subject. It is noteworthy that luster 3 is most significantly enriched with 70% of the BLU factor genes (*p*<0.05; Fisher's exact test). This is in strong concordance with the BLU gene expression signature being highly correlated with temporal disease progression (Figure 3A and 3B). Furthermore, 90% of “acute respiratory viral” signature genes are found in cluster 3 (Table S5) [17]. In comparison, the lack of symptoms in Asx subjects was consistent with their nearly-constant low-level expression of this same cluster of genes (Figure 3B). Interestingly, the two largest clusters, cluster 4 (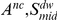) and cluster 6 (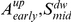), were the most negatively correlated with the development of symptoms, (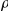 = −0.54) and (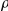 = −0.41) respectively (Figure 3C). These demonstrate the close association between the host transcriptional signatures and the overt clinical disease development.

### Cluster 6 contains significant proportion of genes encoding influenza virus interacting proteins

A recent study identified 66 and 87 human proteins that physically interact with H3N2/Udorn and H1N1/A/PR/8/34 (PR8) viruses, respectively [25]. We examined the distribution of genes corresponding to these proteins among the eight clusters identified in our analysis. Several interesting findings result from the comparison. A total of 27 (45%) and 40 (46%), respectively, of genes overlap with the set of differentially expressed genes found in our study (Figure 3D). The majority of these genes (67%) are found in cluster 4 and 6. Except for clusters 2 and 3, the H3N2/Udorn and H1N1/PR8 genes are distributed in a similar proportion across the eight SOM clusters. Such similarity shows functional conservation between the two viral strains. Secondly, cluster 6 alone contains 44% of the 27 overlapping genes (H3N2/Udorn). This is significantly disproportional to the size of cluster 6 (*p*-value<0.05; Fisher exact test). Several of the overlapping genes such as PRKRA, MAPK9, and NRF1 have been shown to play important roles in host immune or antioxidant function. Thirdly, cluster 2 and 3 showed a significantly lower proportion of overlapping genes (*p*-value<0.05; Fisher's exact test). These results suggest that genes in these two clusters are more likely to be indirectly regulated by the viruses such as those involved in inflammatory responses. Taken together, the results independently validate the functional relevance of the molecular signatures identified in our challenge study and suggest that many cluster 6 genes might be directly regulated by viruses.

### The host antiviral program is activated 36 hours before peak symptom time

**Figure 4:**
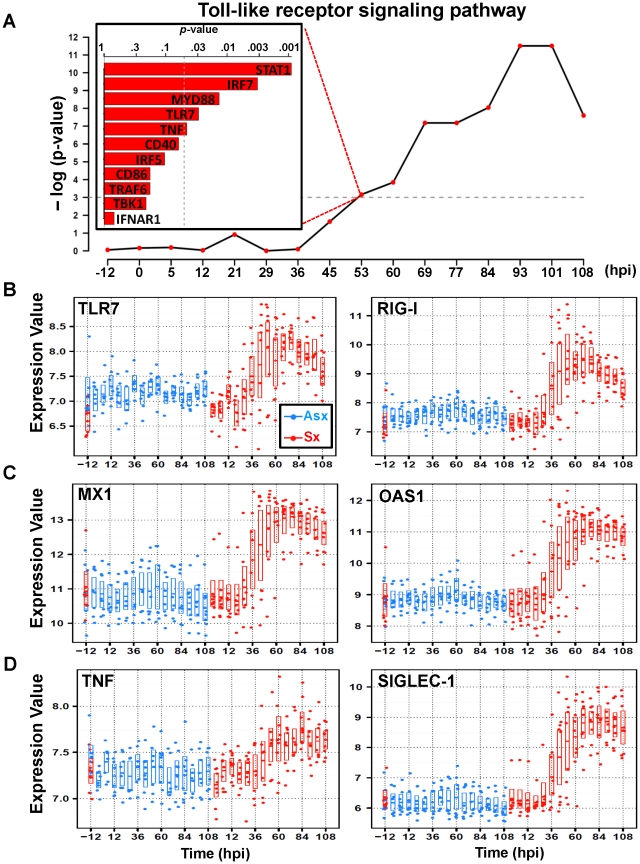
Similar expression dynamics of TLR7-pathway effector genes in cluster 3. (A) Significance of association (*p*-value) between Toll-like receptor (TLR) pathway and overall symptom severity. Significant positive association between TLR-pathway genes and symptom severity is shown at 53 hpi (top left). The temporal expression of representative significant genes on TLR-pathway that are related to pattern recognition (TLR7 and RIG-I) (B); antiviral: myxovirus resistant 1 (MX1) and 2′,5′-oligoadenylate synthetase 1 (OAS1) (C); and pro-inflammatory: TNF and SIGLEC-1 (D). The expression intensities are plotted on a log base 2 scale and all genes are differentially expressed between Asx and Sx (*q*-value
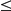
0.0001; EDGE).

An examination of the highest ranked genes in cluster 3 (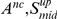) reveals strong activation of host antiviral defense program (Table 1). These genes include several PRR genes such as Toll-like receptor 7 (TLR7), the RNA helicases (RIG-I), and interferon induced with helicase C domain 1 (IFIH1) – genes encodes proteins that are key to innate immune responses by acting as viral RNA sensors [12], [26]–[28]. These are among the most statistically significant (*q*-value<0.0001; EDGE), exhibiting dramatic increase of expression starting at 45 hpi in Sx hosts (Figure 4B, Figure S8). Previous studies have demonstrated that the downstream signaling triggered by these PRRs converge at TANK-binding kinase 1 (TBK1), resulting in direct phosphorylation of interferon regulatory factor 7 (IRF7) [29]. Both TBK1 and IRF7 (Figure S1) have similar expression dynamics and are found in cluster 3. In total, cluster 3 contains 11 genes from the TLR signaling pathway, including MyD88, TRAF6, and STAT1. As a group, they showed an aggregated effect that is significantly associated with the symptomatic disease. This association reaches statistical significance (p<0.05; Globaltest) at 53 hpi with an increasing trend appearing as early as 36 hours before peak symptom time. By 93 hpi, the association attains its maximum level of significance with all 11 member genes significantly upregulated (Figure 4A, Figure S1).

The activation of PRRs by viral ligands triggers downstream signaling cascades that include both antiviral and inflammatory responses. In line with this, cluster 3 contains many such downstream effector genes that were fully activated with similar dynamics. Several interferon-stimulated antiviral genes, such as MX1, OAS1, RSAD2, PKR, exhibit Sx-specific significant temporal activation beginning at 36–45 hpi (Figure 4C, Figure S3, Figure S9). This increase persists many hours beyond symptom peak time, suggesting non-rescinding efforts in viral resolution by the host. It is noteworthy that none of the type-I interferon genes themselves is differentially expressed between the Sx and Asx phenotypes. Similarly, cluster 3 also contains many elements of the inflammatory branch of TLR signaling, e.g., the interferon regulatory factor 5 (IRF5). As a master regulator of the inflammatory arm of TLR7 signaling [9], IRF5 directly activates proinflammatory cytokine tumor necrosis factor alpha (TNF), which has been directly implicated in flu-like symptoms in many types of diseases with excessive inflammation. These and other mediators of inflammatory response such as IL15 and IL10 genes share similar Asx-specific increasing pattern (Figure 4D, Figure S7). Of interest, the sialic acid binding Ig-like lectin 1 (SIGLEC1 or Sialoadhesin) was strongly activated in Sx hosts at mid-to-late stage of infection (Figure 4D). As a macrophage-specific adhesion molecule, SIGLEC1 has recently been related to pro-inflammatory function of macrophages in HIV infections [30]. These results show that the expression kinetics of cluster 3 genes constitutes a transcriptional signature of host antiviral program. This signature fully presents itself 36 hours before the peak symptom time and it is indicative of disease severity. Moreover, its activation intensity maintained high level through 108 hpi.

### An active asymptomatic state is characterized by down-regulated expression of the NLRP3 inflammasome, CASP5, and the IL1B pathway

Members of cytoplasmic Nod/NACHT-LRR (NLR) family have recently been linked to pathogen pattern recognition. Originally identified in bacterial infections, this family of molecules is important to the function of innate immunity [31]–[33]. A recent study showed that nucleotide-binding oligomerization domain 2 (NOD2) recognizes ssRNA of both Influenza and respiratory syncytial viruses [34]. Furthermore, activated NODs were linked to the activation of receptor-interacting serine-threonine kinase 2 (RIPK2) and subsequently nuclear factor kappa-B (NFkB) activation whereas activated NLPRs result in forming so-called inflammasome complexes. This process involves caspase-1 (CASP1) and caspase-5 (CASP5) and ultimately the release of pro-inflammatory and pro-oxidant cytokine interleukin 1-beta (IL1B) [35], [36].

**Figure 5:**
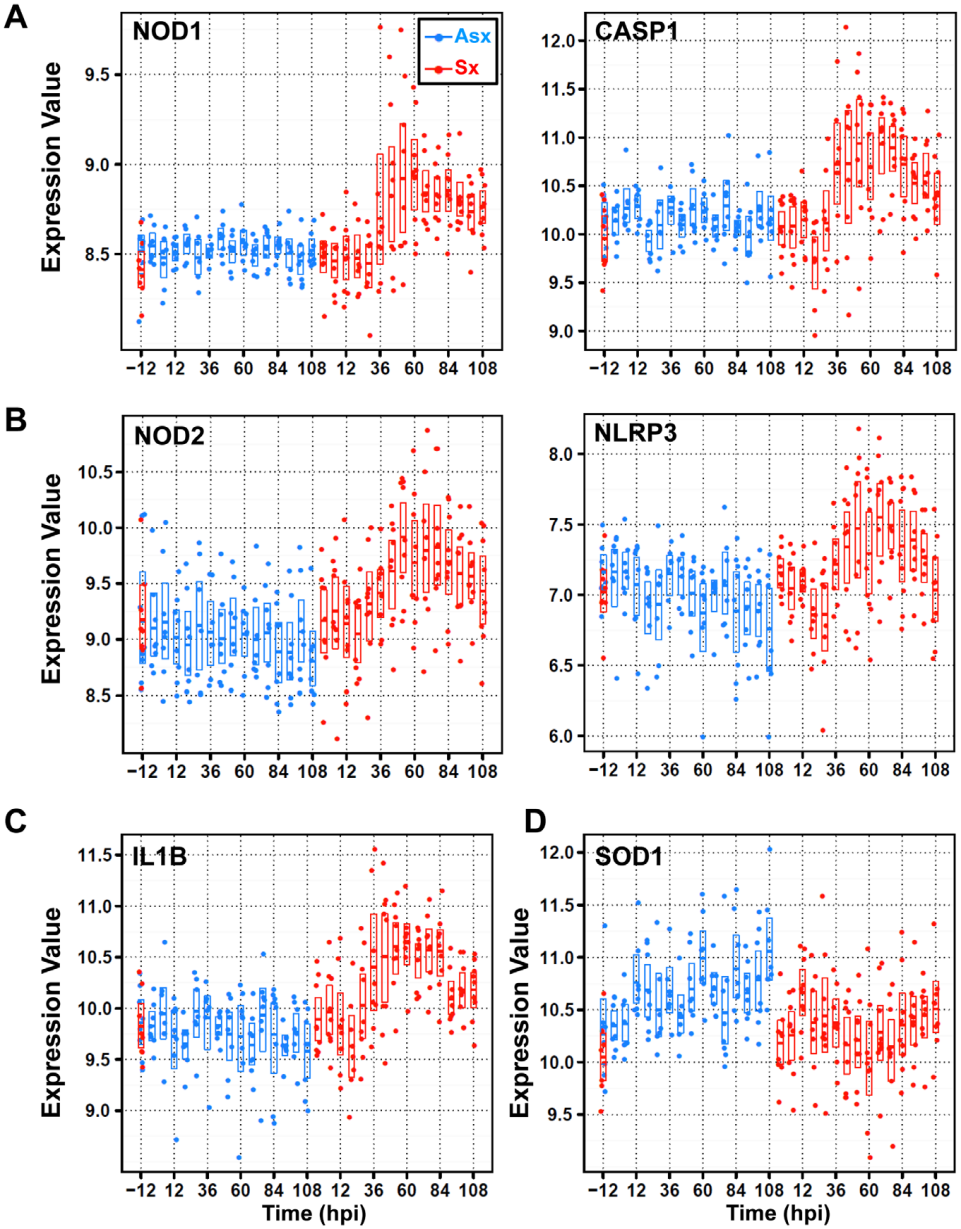
Divergent expression patterns of Nod/NACHT-LRR (NLRs) family of genes from cluster 2 and cluster 3 with contrasting expression of anti-oxidant/stress genes SOD1 and STK25 (or SOK1). (A) SOM cluster 3 genes nucleotide-binding oligomerization domain containing 1 (NOD1) and caspase 1 (CASP1) display strong temporal upregulation in symptomatic subjects. (B) SOM cluster 2 genes NOD2 and NLRP3 exhibit downregulation in Asx hosts and upregulation in symptomatic subjects. (C) SOM cluster 2 gene interleukin 1 beta (IL1B) shows symptomatic-specific upregulation versus Asx-specific downregulation over time. (D) SOM cluster 6 genes superoxide dismutase (SOD1) shows upregulation versus downregulation in Asx and Sx hosts, respectively. The expression values are plotted on a log base 2 scale and all genes are significantly differentially expressed between Asx and Sx (*q*-value
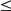
0.0001; EDGE).

The NLR-related genes are among the most highly differentially expressed genes discovered in our study. These genes appear in two clusters, cluster 2 (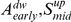) and cluster 3 (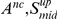), exhibiting markedly different temporal patterns (Figure 2). Residing in cluster 3, NOD1, RIPK2 and CASP1 showed no significant change in Asx subjects (*q*-value>0.01; EDGE) but highly increased among Sx individuals (*q*-value<0.0001; EDGE) (Figure 5A, Figure S2). On the other hand, NOD2, NLPR3, and CASP5 are found in cluster 2. Their expression decreased in Asx but increased evidently in Sx (Figure 5B, Figure S2). In addition, the expression level of IL1B (cluster 2) was evidently suppressed in the Asx phenotype while activated in the Sx phenotype (Figure 5C). Given the importance of NOD2 and NLPR3 to the processing of IL1B, the Asx specific lower expression of IL1B may be contributed directly to the similar downregulation patterns of NOD2 and NLRP3. This hypothesis is supported by a new study in which Nod2-deficient mice showed decreased levels of TNF and IL1B in PBMC [34].

Of relevance to the phenotypically different expression dynamics of NLR-mediated inflammasome activation, an opposite trend is observed in two cluster 6 (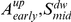) genes that are related to cellular response to oxidative stress. The superoxide dismutase (SOD1) and serine/threonine kinase 25 (STK25 or SOK1) are markedly activated in Asx subjects, contrasting to the transient suppression pattern (45–60 hpi) in Sx hosts (Figure 5D, Figure S10). As SOD1 and STK25 both have been linked to anti-oxidant/stress response and reduced concentration of ROS [37]–[39], their sustained up-regulation in Asx hosts highlights a host response signature unique to the Asx phenotype. This signature may relate to the concomitant suppression of NLRP3 and IL1B in Asx individuals. Collectively, our data reveal a phenotypically divergent expression of NLR family genes and inflammasome signaling, which may be related to the host anti-oxidant response.

### Distinct temporal kinetics of JAK-STAT pathway and SOCS family genes reveals a potential anti-inflammatory and viral control mechanism in Asx hosts

**Figure 6:**
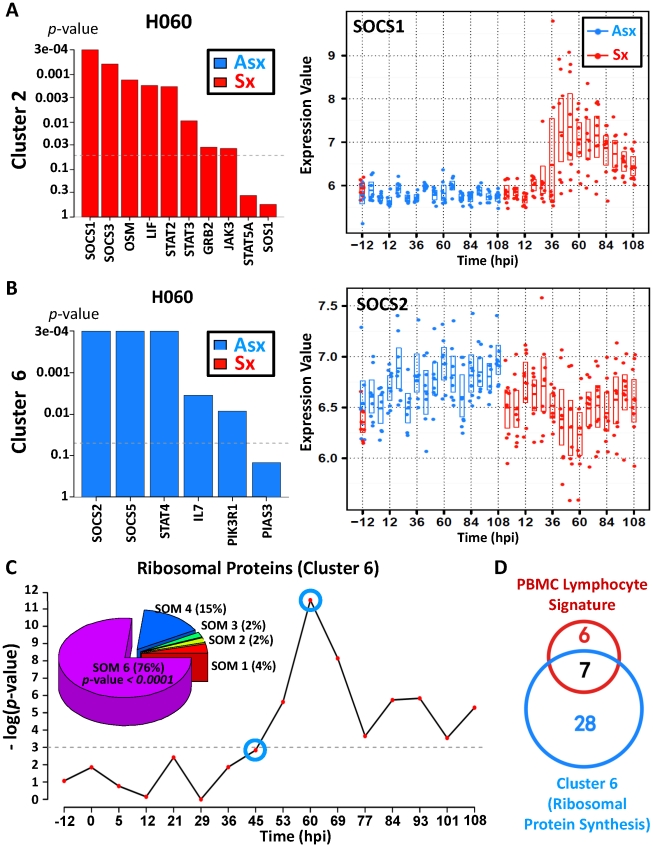
Asymptomatic hosts showed unique temporal expression kinetics of cluster 6 genes related to JAK-STAT signaling transduction and protein biosynthesis. (A,B) Distinct expression pattern of gene members in JAK-STAT pathway and their association with symptom severity. (A) Significant positive association between genes and disease severity is shown for 60 hpi (left); temporal gene expression pattern of suppressor of cytokine signaling 1 (SOCS1) shows upregulation in symptomatic hosts. (B) Significant negative association between genes and disease severity is shown for 60 hpi (left); temporal gene expression pattern of SOCS2 shows upregulation in Asx hosts versus downregulation in Sx hosts. (C) Significance of negative association (*p*-value) between ribosomal protein synthesis (RPS)-related genes and overall disease severity; Pie chart (top left) shows a high degree of enrichment of significant RPS genes in SOM cluster 6, which is characterized by a trend of upregulation (in Asx hosts) versus downregulation (in symptomatic hosts) over time. (D) Proportion overlap between cluster 6 ribosomal protein synthesis genes and lymphocyte signature ribosomal proteins genes [49].

A hallmark of host recognition of viral RNA is the activation of Janus kinase-signal transducer and activator of transcription (JAK-STAT) pathway, which is crucial for the antiviral function of interferons. However, such activation is tightly controlled to limit the possibility of over-stimulating inflammatory cytokine-receptor signals. As an integral component of the JAK-STAT pathway, the family of suppressor of cytokine signaling (SOCS) proteins have recently been reported to negatively regulate the response of immune cells to cytokine signals [40]. Using pathway analysis, we detected significantly distinct JAK-STAT signaling dynamics (*p*-value<0.05; Globaltest), involving two different sets of SOCS genes. The first set included SOCS1 and SOCS3 from cluster 2 (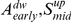) while the second group consists of SOCS2 and SOCS5 from cluster 6 (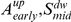). The expression of SOCS1 and SOCS3 declines at early time points among Asx but strongly increases among Sx (Figure 6A, Figure S11). Growing evidence suggests that SOCS1 and SOCS3 are important inhibitory modulators in limiting the inflammatory effect of interferon signaling during viral infection [41], [42]. Our data supports such a protective role of SOCS1 and SOCS3 given their much higher levels of expression during late infection phase (45 hpi onward).

Consistent with cluster 6 but contrasting with the cluster 2 expression pattern (Figure 2), SOCS2 and SOCS5 exhibits expression dynamics that clearly differ from that of SOCS1 and SOCS3. Starting from the early infection stage (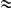
12 hpi), SOCS2 and SOCS5 show marked increasing trend in Asx and this trend persists throughout the entire infection period (Figure 6B, Figure S11). In contrast, their expression diminishes in Sx, especially between 45 hpi and 69 hpi. A recent study showed that the anti-inflammatory actions of aspirin-induced lipoxins depend upon the function of SOCS2 [43]. Highly expressed in lymphoid organs, SOCS5 was hypothesized to be important for the generation of Th1 responses by repressing IL-4-induced signals that promote Th2 differentiation [44]. In addition, we observed a significant positive association of interleukin 7 (IL7) and STAT4 (Figure 6B). Of these, STAT4 transduces IL12 and IFN-A cytokine signals in T cells and monocytes [45] whereas IL7 is critical for proper T cell response and expansion during viral infection [46]–[48]. Taken together, the distinct expression patterns of SOCS family genes and related JAK-STAT signaling suggest possible early involvement of Th1-type adaptive immune response in Asx hosts with no sign of excessive inflammation.

### Ribosomal protein synthesis genes are upregulated in Asx subjects as compared to Sx subjects

In addition to expression changes in magnitude, genes in clusters 2 (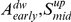) and 6 (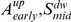) also exhibit directional contrast between two phenotypes. As the largest cluster with a total of 1,326 member genes, cluster 6 contains genes with expression profiles similar to those of SOCS2 and SOCS5. Among them, we found an unusual saturation of genes related to ribosomal protein synthesis. Out of 47 significant genes in this pathway, 35 (76%) of them are located in cluster 6 (*p*-value*<0.0001*; 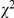
test). Together, these 35 genes correlated positively with Asx phenotype (*p*-value<0.05; Globaltest) and their expression increased over the course of the study (Figure 6C). Such association emerges at 45–53 hpi and peaks at 60 hpi, at which point every one of the 35 genes becomes highly expressed. Individually, all genes showed increased expression trend (Figure S4). This trend can be seen at as early as 5 hpi and as late as 108 hpi. In contrast, Sx subjects showed sustained down-regulation of the same set of genes, with lowest expression level at 60 hpi. This decreasing trend continues until ∼84 hpi, which coincides with the peak symptom time observed in symptomatic subjects (Figure 3). In addition, these 35 genes include 53% of 13 genes whose expression are characteristic of peripheral blood lymphocytes (Figure 6D) [49], suggesting prominent presence of lymphocytes in the blood of Asx subjects during infection. This is further supported by the increased number of whole blood leukocytes in Asx subjects (Figure S17). Given the markedly contrasting trends observed between Asx and Sx phenotypes, we conclude that Asx hosts responded differently to the viral insult by inducing leukocyte response with enhanced cellular protein biosynthesis.

## Discussion

Pathogenic influenza A viral infection is a complex and dynamic process that involves various components of the host immune system at different stages of infection in response to virus-induced physiological changes. Dissecting the temporal host response to invading viruses and subsequent symptomatic disease process are crucial for studying disease pathogenesis and related host factors. Equally important is to understand the complexity of the host response in individuals who are exposed but effectively contain the infection and avoid symptomatic disease. This study presents key transcriptional differences between Asx and Sx host responses, and highlights an active state (on a gene transcription level) of viral control in both Sx and Asx hosts.

### Viral sensing, inflammation, and symptomatic disease

We showed that the viral sensing and inflammation in Sx hosts clearly correlate to clinical symptom development over time. As mounting evidence has established the role of various PRRs in sensing viral components of influenza viruses, our results confirm the concurrent activation of all known classes of PRRs and their signaling cascades by influenza viruses in human challenge models. In contrast, Asx hosts showed not only an absence of such activation, but also negative regulation of related inflammatory signals, especially in the case of NLRP3 and NOD2. This corresponds to their lack of clinical apparent symptoms.

It has long been postulated that multiple PRRs represent a functional redundancy of host defense and that there exists signaling crosstalk among them, stimulating similar cytokine profiles that are both pro-inflammatory and pro-oxidant [36]. Here we found simultaneous and continued activation of all known PRRs in Sx hosts with particular emphasis on NLR family genes. Of important relevance, two recent studies showed that H1N1 1918 pandemic virus induced upregulation of inflammasome components in a macaque model while avian H5N1 virus Vietnam/1203/04 caused increasing expression of NLR family genes in mice [50], [51]. In both cases, the early and sustained upregulation of inflammasome component genes was directly associated with lethal or detrimental host responses. Abnormal expression of NOD2 has been implicated in inflammatory bowel disease [52], [53]. Conversely, it was shown in a study on chronic arthritis that Nod2 gene-deficiency resulted in reduced joint inflammation and increased protection against early cartilage damage in mice [54]. Our results provide new evidence for a much broader role played by NLR-family genes during influenza viral infection that is likely to be shared by multiple viral strains and influenced by specific cellular context. Their contrasting expression dynamics in Sx versus Asx points to potential benefit in controlling inflammation by regulating NLRP3-mediated inflammasome activation or other inflammatory responses [55].

### Link between anti-oxidant response and Asx infection

The inflammasome and pro-inflammatory cytokines have been linked to increased level of oxidative stress during viral infection [56]–[58]. A recent report showed in mouse model that Nlrp3 inflammasome activation depends on reactive oxygen species (ROS) and inhibition of ROS induction abolished IL1B production during influenza infection [59]. It is intriguing that our data shows a temporal Asx-specific upregulation versus Sx-specific suppression of SOD1 and SOK1. This coincides with the observed negative correlation between these genes and NLRP3. Since SOD1 and SOK1 are capable of reducing ROS and of suppressing oxidative stress [37], their increased expression in Asx hosts may play a role in negatively regulating NLRP3 expression and inflammasome signaling. In support of this hypothesis is a study on the efficacy of antioxidant therapy found that pyran polymer-conjugated SOD1 protected mice against potentially lethal influenza virus infections [38]. Together, our results provide evidence for a protective role of antioxidants SOD1 and SOK1. Their increased mRNA expression may constitute an effective antiviral mechanism by which aberrant immune responses are avoided in Asx hosts.

### The nature of Asx phenotype

It is estimated that Asx infections account for 30–50% of seasonal flu cases [2], which is consistent with the attack rate in our study. Since both Asx and Sx subjects were challenged under the same protocol and displayed inoculation dosage-independent viral shedding, this raises a critical question concerning the nature of the observed Asx phenotype. We have strong evidence that the observed Asx molecular signatures are a consequence of rapid innate response rather than being due to failed inoculation. Firstly, 50% of Asx subjects had evident viral shedding. This is on par with that of “subclinical” or “secondary” infections reported in the literature. In addition, serum neutralizing antibody (nAb) titre were nearly identical in Asx and Sx subjects on day 0 and day 7 with pre-inoculation nAb independent of disease severity. Critically, the nAb titre increased over time in both Asx and Sx individuals (Figure S12). This indicates a boosting effect of immunity, and suggests that even if viral replication was inhibited, enough viruses were detected by the Asx host immune system to cause expansion of Ab producing cells. Secondly, there was no apparent dosage effect – subjects who received relatively lower amount of inoculation do not necessarily become more ill than individuals who received higher dose of virus. We found no statistically significant dependence between disease outcome and inoculation dosage (Figure S13A). Furthermore, the amount of viral shedding from the site of infection did not appear to differ among groups who received varying inoculation doses (Figure S13B). Thirdly, Asx subjects presented dramatic transcriptional responses towards inoculation. When their expression profiles were studied alone, more than 3,000 genes showed significant post-infection expression changes. These changes do not correlate with the amount of virus detected. Two subjects (#3 and #17) who never yielded detectable virus (<1.25 TCID_50_/mL) in their nasal wash appeared to have the most significant temporal suppression of gene NLRP3 (Table S2; Figure S14; Figure S15). Additionally, the responses of two seroconverted Asx subjects (#2 and #3), according to haemagglutination inhibition (HAI) assay, are not different from those of other Asx individuals (Table S2; Figure S14; Figure S15).

With all presented evidence supporting the activation of a robust Asx immune response, our findings point to an important host factor that may lead to such Asx subclinical infections. Shutting down protein synthesis helps control infection by inducing apoptosis of infected cells [60]–[62]. Consistent with this, we observed marked downregulation of protein biosynthesis and apoptosis related genes in Sx hosts at mid-to-late stages (Figures S4, S5, S6). A similar lowering expression of ribosomal proteins has been reported in measles-infected dendritic cells [63]. What is surprising is the sustained upregulation of as many as 35 ribosomal proteins in only Asx subjects (Figure 6C, Figure S4). The increased ribosomal gene expression has been associated with peripheral blood lymphocytes [49] and our data also showed significant increase of white blood cells in Asx subjects (Figure S17). Lacking strong PRRs activation, and hence an absence of adaptive immune response, these Asx hosts appeared to be capable of mounting a more potent cell-mediated innate immune response than the symptomatic subjects.

### Uncontrolled factors

As our study mainly focuses on gene expression in whole peripheral blood, it is possible that the changes observed in gene expression levels are at least partially due to changes in cell population. However, this is unlikely for two reasons. First, the maximum observed change in cell populations for both Asx and Sx hosts was no more than 80% from baseline (Figure S17). Second, the distribution of leukocyte subpopulations is not correlated with phenotype at baseline or throughout the time course of the study (Table S6). Thus, the dramatic changes in gene expression described here cannot be attributed greatly to cell population changes. Another uncontrolled factor is that certain subjects may have come into the study with related preconditions. While we cannot completely dismiss the possibility of previous exposure to other respiratory viruses, all subjects were healthy and tested negative for H3N2 influenza antibody at pre-inoculation time. None of the volunteers had been vaccinated for any influenza virus in the previous 3 years. Finally, while we did not observe subject demographics such as age, gender, or ethnicity to be influential of final disease outcome (Table S1b), we cannot rule out the possibility of small sample bias. We have been careful to provide statistical safeguards against model overfitting by reporting significance measures (*p*-values and *q*-values with qualifying confidence intervals) that are associated with our findings.

### Concluding remarks

To our knowledge, this multi-institutional collaborative study presents the first systematic analysis of the full temporal spectrum of pathogen-elicited host responses during influenza viral infection. This work represents by far the most extensive *in vivo* human challenge study on influenza viruses. Combined with key clinical parameters, our results offer an opportunity to look beyond individual signaling events and into their collective effects on symptomatic disease pathogenicity. The detailed timing of various immune response events *in vivo* will advance our understanding of their biological and clinical relevance to influenza virus-mediated disease progression.

## Materials and Methods

### Human influenza viral challenges

We performed a healthy volunteer dose-ranging intranasal challenge with influenza A A/Wisconsin/67/2005 (H3N2) at Retroscreen Virology, LTD (Brentwood, UK). We enrolled 17 pre-screened volunteers aged 18 to 45 years of age who provided informed consent. All volunteers were without recent influenza-like illness in the preceding 45 days, tested influenza A H3N2 antibody negative by HAI at pre-inoculation screening and had not been vaccinated with a seasonal influenza vaccine within the preceding 3 years. On day of inoculation, a dose of 10^6^ TCID_50_ Influenza A manufactured and processed under current good manufacturing practices (cGMP) by Bayer Life Sciences (Vienna, Austria) was inoculated intranasally per standard protocol at a varying dose (1∶10, 1∶100, 1∶1000, 1∶10000) with four to five subjects receiving each dose. Subjects were not released from quarantine until after the 216th hour. Blood and nasal lavage collection continued throughout the duration of the quarantine. All subjects received oral oseltamivir (Roche Pharmaceuticals) 75 mg by mouth twice daily prophylaxis at day 6 following inoculation. All patients were tested negative by rapid antigen detection (BinaxNow Rapid Influenza Antigen; Inverness Medical Innovations, Inc) at time of discharge. All exposures were approved by the relevant institutional review boards and conducted according to the Declaration of Helsinki.

### Case definitions

Symptoms were recorded twice daily using standardized symptom scoring [2]. The modified Jackson Score requires subjects to rank symptoms of upper respiratory infection (stuffy nose, scratchy throat, headache, cough, etc) on a scale of 0–3 of “no symptoms”, “just noticeable”, “bothersome but can still do activities” and “bothersome and cannot do daily activities”. For all cohorts, modified Jackson scores were tabulated to determine if subjects became symptomatic from the respiratory viral challenge. A modified Jackson score of > = 6 over the first five days period was the primary indicator of successful viral infection [18], [64] and subjects with this score were denoted as “Symptomatic” (Sx). Viral titers from daily nasopharyngeal washes were used as corroborative evidence of successful infection using quantitative PCR (Table S2) [18], [64], [65]. Subjects were classified as “Asymptomatic” if the Jackson score was less than 6 over the first five days of observation and viral shedding was not documented after the first 24 hours subsequent to inoculation. Successful inoculation in Asx hosts was further validated by analysis of multimodal data including serum neutralizing antibody and haemagglutination inhibition titers. For additional evidence see discussion in Text S1. Standardized symptom scores were tabulated at the end of each study to determine attack rate and time of maximal symptoms (time “T”). The clinical disease is mild (only a single fever was observed). Immune activation assays (such as antibody response) over the full time course of the challenge study were not available for our analysis.

### Biological sample collections

During the challenge study, subjects had samples taken 24 hours prior to inoculation with virus (baseline), immediately prior to inoculation (pre-challenge) and at set intervals following challenge: peripheral blood for serum, peripheral blood for RNA PAXgene™, nasal wash for viral culture/PCR, urine, and exhaled breath condensate. Peripheral blood was taken at baseline, then at 8 hour intervals for the initial 120 hours and then 24 hours for the remaining 2 days of the study. For all challenge cohorts, nasopharyngeal washes, urine and exhaled breath condensates were taken at baseline and every 24 hours. Samples were aliquoted and frozen at −80°C immediately.

### RNA purification and microarray analysis

RNA was extracted at Expression Analysis (Durham, NC) from whole blood using the PAXgene™ 96 Blood RNA Kit (PreAnalytiX, Valencia, CA) employing the manufacturer's recommended protocol. While whole blood RNA is initially extracted, a secondary procedure (B-globin reduction) was then employed to remove the contribution of red blood cell (RBC) RNA to the total RNA. A set of four peptide nucleic acid (PNA) oligomers whose sequences are complementary to the 3′ portions of the alpha and beta hemoglobin RNA transcripts were added to reduce globin RNA transcription due to RBC. The inhibition of globin cDNA synthesis dramatically reduces the relative amount of anti-sense, biotin-labeled cRNA corresponding to the hemoglobin transcripts. Hybridization and microarray data collection was performed using the Human Genome U133A 2.0 Array (Affymetrix, Santa Clara, CA) and expression profiles were pre-processed using robust multi-array (RMA) method [66] (Text S1). Both raw and normalized gene expression data are available at GEO (GSE30550).

### Statistical analysis

Temporal gene expression was analyzed using EDGE [20] on RMA normalized intensities. A total of 5,076 genes were identified as most significantly differentially expression genes (*q*-value<0.01) between Asx and Sx. Co-clustering of the significant genes found by EDGE was performed using Self-Organizing Map [21] (Text S1). We estimated the correlation between disease symptom scores and temporal expression values of clusters using a standard linear mixed model [67], [68]. Specifically, for each individual symptom measured, we regressed the scores onto the expression value vector of each SOM cluster, separately, with a random-effects term accounting for within-subject temporal correlation. Biological pathway enrichment analysis was performed using Ingenuity Pathway Analysis (IPA). We implemented the non-parametric Jonckheere-Terpstra (JT) method [69] to test monotonicity of the expression patterns of individual gene clusters. Briefly, the JT test was applied independently to each cluster and configured to test the null hypothesis that there exists no monotonic trend in the temporal change of gene expression. This test was performed separately for each one of two phenotypes separately. The resulted 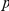-values were adjusted for multiple comparisons with Benjamini-Hochberg method [70].

To identify canonical gene pathways in each SOM cluster that are highly associated with disease phenotypes, we applied Globaltest [71] using the pathway definition in MsigDB database (v2.5) [72] that include both pathway components and targets. We assessed the correlation between clinically determined symptom scores and the temporal gene expression of SOM clusters using standard linear mixed model regression. The correlation (R value) was estimated using a *signed coefficient of determination* [67], [68].

The BLU factor analysis was used to detect disease signature shown in Figure 1A. Unlike our implementation of EDGE, SOM and Globaltest, BLU is an unsupervised method requiring no prior class information. Like other unsupervised Bayesian factor analysis methods, BLU finds a decomposition of the data matrix **Y**, here a ***p*** by ***n*** matrix of abundances of the ***p*** mRNA transcripts for each of ***n*** gene expression profiles, into a matrix product **MA** where each column of **M** is a factor and each column of **A** is a set of factor loadings corresponding to individual factors in **M** for a given chip:
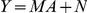
In essence, BLU estimates two matrix valued latent variables **M** and **A**, whose product best approximates the most important information contained in the observation **Y** while minimizing the residual model fitting error (denoted as **N** in the formula above) with latent variable order selection according to an hierarchical Bayesian model. However, unlike other factor analysis, BLU decomposes the data into relative proportions such that the columns of **M** and the columns of **A** are non-negative and the columns of **A** sum to one. Intuitively, a BLU-discovered factor can be viewed as a gene expression profile, whose amplitudes represent the relative contribution of each gene present in that factor, and the factor loadings are the proportions of these factors that are present in each chip. Such positivity constraints aid in interpretation and are natural in gene microarray analysis as the expression intensity measurements of genes are always non-negative.

BLU was run on all genes on the expression array and extracted a total of three major BLU factors. The factor scores of the samples were subsequently divided into two groups: samples taken before inoculation (pre-inoculation samples) and samples taken after inoculation (post-inoculation samples). We then tested for significant difference between the scores of the pre-inoculation and post-inoculation samples (*t*-test with *p*-value less than 0.01). At this significance level only one of the factors passed this test – the acute respiratory factor shown in Figure 1A. Based on the score of this acute respiratory factor, we quantitatively determine the four regions by a threshold criterion using the pre-inoculation samples. The threshold was set to be more than 4 times the maximum pre-inoculation sample score (corresponding to a *t*-test *p*-value less than 0.05) (Text S1). In this manner, all samples were labeled with one of four classes, namely classes 1–4 (Figure 1A).The class designation of a sample indicates distinct risk levels of four intrinsic disease states – uninfected (class 1), infected with low-risk for symptom development (class 2), infected with high-risk for symptom development (class 3), and infected with overt symptoms (class 4).

The genes exhibiting largest contrast between each pair of classes were extracted from all genes on the expression array using a LogitBoost classifier [73] as a contrast function. Note that our objective is not to obtain a classifier between regions but rather to use LogitBoost to identify groups of genes most associated with differences between a pair of classes. As it uses boosting algorithm to perform variable selection, our implementation of LogitBoost yields a set of genes in addition to a classifier function. To do this, we generated 200 bootstrap samples from each class [74]. We randomly selected 2/3 of each bootstrap sample to construct the boosting ensemble and the other 1/3 of data was used to evaluate the variability of the association between the largest contrast genes and each class pair. We defined the largest contrast genes as the set of genes that were selected by LogitBoost algorithm for each class pair more than 100 (50%) of the 200 bootstrap samples. The average expression of these genes are shown in Figure 1C.

## Supporting Information

Figure S1 Temporal expression of Toll-like receptor 7 pathway member genes. Accompanying Figure 2c, temporal expression are shown for TLR7-pathways genes (n = 11) including STAT1, IRF7, MyD88, TLR7, TNF, CD40, IRF5, CD86, TRAF6, TBK1, and IFNAR1. The expression intensities are averaged over subjects in Asx and Sx phenotypes and plotted on a log base 2 scale.
(PDF)

Figure S2 Temporal expression of NLR family genes. 1) cluster 7 gene PYD and CARD domain containing (PYCARD or ASC); 2) cluster 3 gene receptor-interacting serine-threonine kinase 2 (RIPK2); 3) cluster 2 gene caspase 5 (CASP5). The expression intensities are plotted on a log base 2 scale.
(PDF)

Figure S3 Increased temporal expression of antiviral RNA-dependent eIF-2 alpha protein kinase (EIF2AK2 or PKR) in cluster 3. The expression intensities are plotted on a log base 2 scale.
(PDF)

Figure S4 Phenotypically contrasting expression dynamics ribosomal protein synthesis-related genes (n = 35) in cluster 6. The expression intensities are averaged over subjects in Asx and Sx phenotypes and normalized to have zero mean and unit standard deviation.
(PDF)

Figure S5 Symptomatic-specific temporal downregulation of cluster 4 genes (n = 9) that regulate programmed cell death (apoptosis). A) Significance (p-value) of association between phenotypes and the whole group of genes at all time points and at time 45 hpi (top left panel). B) Average temporal expression intensities are computed on subjects in Asx and Sx phenotypes and normalized to have zero mean and unit standard deviation.
(PDF)

Figure S6 Symptomatic-specific temporal downregulation of cluster 4 genes (n = 13) that are related to mitogen-activated protein (MAP) kinase cascades. A) Significance (p-value) of association between phenotypes and the whole group of genes at all time points and at time 45 hpi (top left panel). B) Average temporal expression intensities were computed on subjects in Asx and Sx and normalized to have zero mean and unit standard deviation.
(PDF)

Figure S7 Increased temporal expression of inflammatory response regulators (cluster 3), interleukin 15 and interleukin 10. The expression intensities are plotted on a log base 2 scale.
(PDF)

Figure S8 Temporal gene expression of cluster 3 gene cytoplasmic double-strand viral RNA sensor IFIH1 (interferon induced with helicase C domain 1). The expression intensities are plotted on a log base 2 scale.
(PDF)

Figure S9 Temporal expression of interferon inducible anti-viral genes from cluster 3. The expression intensities are plotted on a log base 2 scale.
(PDF)

Figure S10 Temporal gene expression of cluster 6 gene serine/threonin kinase 25 (STK25 or SOK1). The expression intensities are plotted on a log base 2 scale.
(PDF)

Figure S11 Temporal expression of genes from the family of suppressor of cytokine signaling (SOCS), including cluster 2 gene SOCS3 and cluster 6 gene SOCS5. The expression intensities are plotted on a log base 2 scale.
(PDF)

Figure S12 Neutralizing antibody (nAb) measure prior to inoculation shows no significant phenotypic difference and is not correlated with disease outcome. A, B) nAb of all subjects at Day 0 (A) and day 7 (B). No difference were observed between Asx and Sx on both days (non-parametric rank test). C) No evident correlation between nAb on Day 0 and maximum Jackson standardized score. A linear regression fit of score on nAb readings is shown in dark black line. Correlation test was performed using Spearman test. D) nAb increased in both Asx and Sx subjects from day 0 to day 28. ★ No sample available on day 28.
(PDF)

Figure S13 The infection outcome and viral load are independent of the dosage of viral inoculation. (A) There is no significant correlation between disease outcome and inoculation dosage (*p*-value = 0.2299; Fisher's exact test). Each bar represents a randomized group of four to five subjects receiving a varying dose of Influenza A virus inoculation on day 0 (Supplementary Materials). Within each group, subjects are divided into clinically determined symptomatic (red) and asymptomatic (blue) subgroups. (B) Viral shedding pattern (Table S2A) does not differ across inoculation dosage groups. All nine symptomatic and four asymptomatic subjects showed shedding ≥1.25. Two asymptomatic shedders (#14 and #16) are in lowest dosage group and the other two (#2 and #4) are in the highest dosage group. The amount of viral shedding are determined from nasal wash obtained daily (Supplementary Methods). Shedding values <1.25 are set to 1.25 in the plot.
(PDF)

Figure S14 Asymptomatic subjects demonstrated non-passive transcriptional response program. As an example, we show a significant temporal expression decrease of the inflammasome related gene NLRP3 in eight individual asymptomatic subjects. Each subpanel depicts the temporal expression of one individual asymptomatic subject. The *y*-axis is the log base 2 signal intensity of NLRP3 and the *x*-axis is the time from −12 hpi to 108 hpi (hour post inoculation). A polynomial fitting of expression values (solid line) was fitted using LOESS model and significance of temporal trend was assessed with EDGE. Subjects #3 and #17 never showed detectable amount of virus (<1.25) in their nasal wash (Table S2).
(PDF)

Figure S15 Serological conversion versus clinical symptom outcome and gene expression. The RPL3 gene expression trajectories for Asx (blue) and Sx (red) are representative of SOM cluster 6. Legend at right gives the character encoding of each subject along with their disease outcome (‘blue’ Asx and ‘red’ Sx) and their serologic conversion outcome (‘+’ converted and ‘−’ not converted). There is no significant relation between disease outcome and serological conversion (*p*-value of 0.27 according to likelihood ratio test of dependency between these two outcomes). The two seroconverted asymptomatic individuals (subject #2 and #3) are called out by orange arrows in the gene expression trajectory plot. The RPL3 expression profiles of these two subjects are not significantly different from those of the other asymptomatic hosts.
(PDF)

Figure S16 Schematic outline of analysis pipeline. Unsupervised: no clinical phenotype information was used. Supervised: clinical phenotype was incorporated in analysis.
(PDF)

Figure S17 Daily white blood cell counts show mild change (less than 80%) from baseline in Asx and Sx phenotypes.
(PDF)

Figure S18 Expression heatmap of genes that are significantly differentially expressed between Asx and Sx. Genes are identified using EDGE (*q*-value<0.01) and clustered with SOM. The average expression are computed and normalized for each gene to have zero mean and unit standard deviation. Within a cluster, genes are shown in decreasing order of significance level.
(PDF)

Table S1 (A) Subject Demographic and Clinical Characteristics of Viral Challenge Cohort. (B) Detailed subject demographics.
(PDF)

Table S2 Viral shedding and serological testing data for all human volunteers (n = 17) challenged with Influenza H3N2 viruses. A) Measure of viral titre isolated from nasal wash over a total of 9 days. B) Serological data on pre-screening, −24 hpi, and +28 days.
(PDF)

Table S3 Significance of monotonic trend of gene expression in SOM clusters. For the genes in each SOM cluster (Figure 1), we implemented the Jonkheere-Terpstra (JT) test (Text S1) of significance on Asx and Sx subjects, respectively, to test for monotonic increase or decrease of gene expression over time. Columns 2 and 3 show *p*-values associated with the null hypothesis that genes in the cluster have no monotonic trend. Red colored entries indicate clusters having highly significant monotonic expression profiles for a particular phenotype.
(PDF)

Table S4 Discriminatory genes selected by each logistic boosting model. Genes are listed in decreasing order based on their discriminatory power in each model.
(PDF)

Table S5 Comparison of genes identified by Zaas *et al* with significant genes in the present manuscript.
(PDF)

Table S6 The proportions of primary white blood cell (WBC) subtypes are similar between Asx and Sx. White blood cells counts were obtained daily through standard laboratory workout. * Phenotype specific average percentage of cell subpopulation were computed using Tukey's biweight robust M-estimator. The null hypothesis *H_0_*: the frequency distribution of WBC subtypes is independent of disease phenotype was performed using Fisher-exact test. *H_0_* is rejected at significance level of 0.01.
(PDF)

Text S1 Supplementary methods and supplementary discussions.
(DOC)
