# Supplementary figures and images for: Temporal Dynamics of Host Molecular Responses Differentiate Symptomatic and Asymptomatic Influenza A Infection
Source: PLoS Genet. 2011 Aug 25;7(8):e1002234. doi: 10.1371/journal.pgen.1002234 (PMC3161909; doi:10.1371/journal.pgen.1002234)

Fig. S1

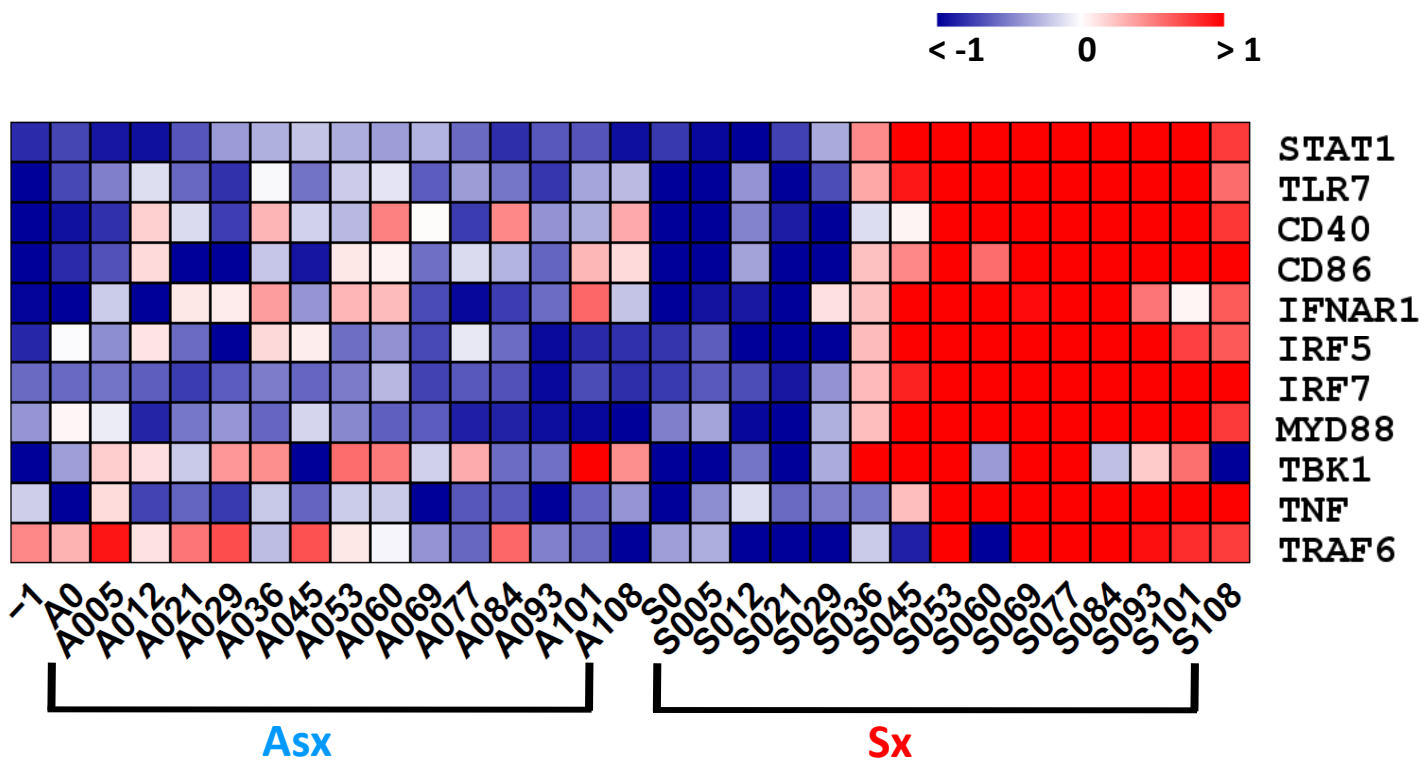

Supplement: Figure S1 — Temporal expression of Toll-like receptor 7 pathway member genes. Accompanying Figure 2c, temporal expression are shown for TLR7-pathways genes (n = 11) including STAT1, IRF7, MyD88, TLR7, TNF, CD40, IRF5, CD86, TRAF6, TBK1, and IFNAR1. The expression intensities are averaged over subjects in Asx and Sx phenotypes and plotted on a log base 2 scale. (PDF) [file pgen.1002234.s001.pdf]

## Fig. S2

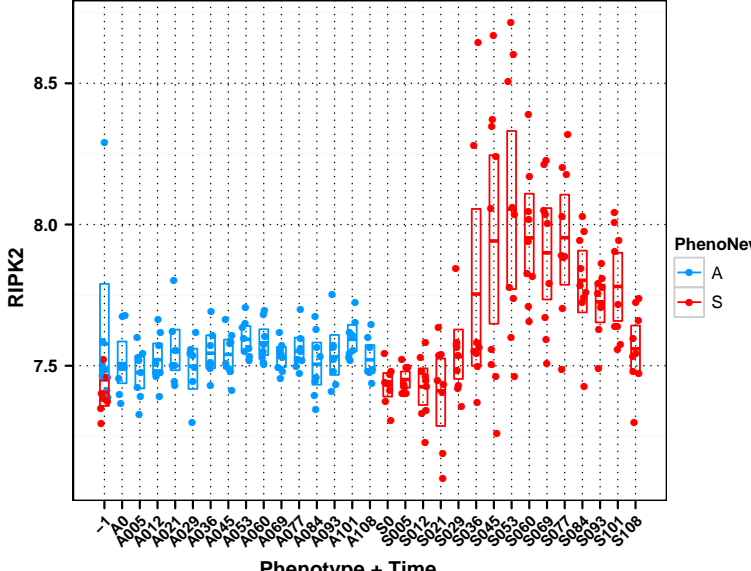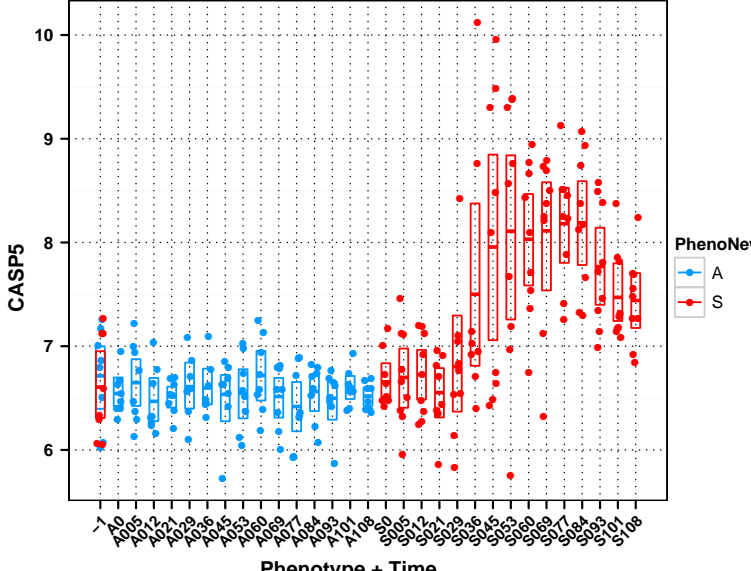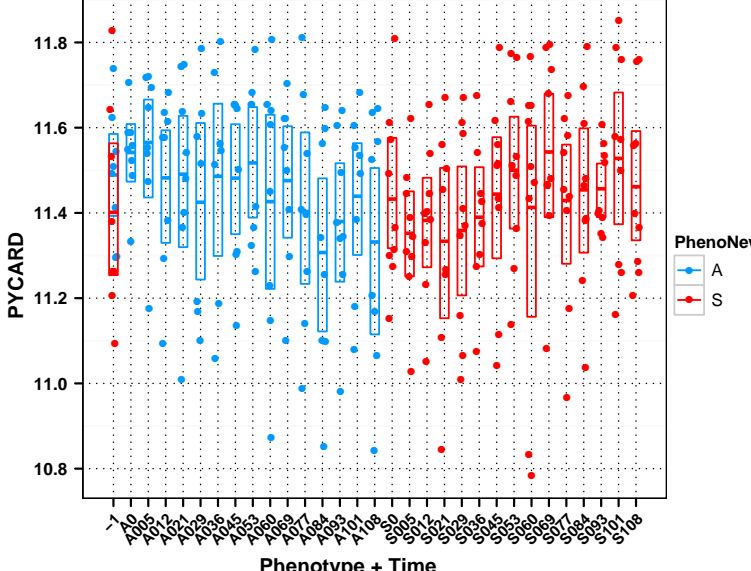

Supplement: Figure S2 — Temporal expression of NLR family genes. 1) cluster 7 gene PYD and CARD domain containing (PYCARD or ASC); 2) cluster 3 gene receptor-interacting serine-threonine kinase 2 (RIPK2); 3) cluster 2 gene caspase 5 (CASP5). The expression intensities are plotted on a log base 2 scale. (PDF) [file pgen.1002234.s002.pdf]

Fig. S3

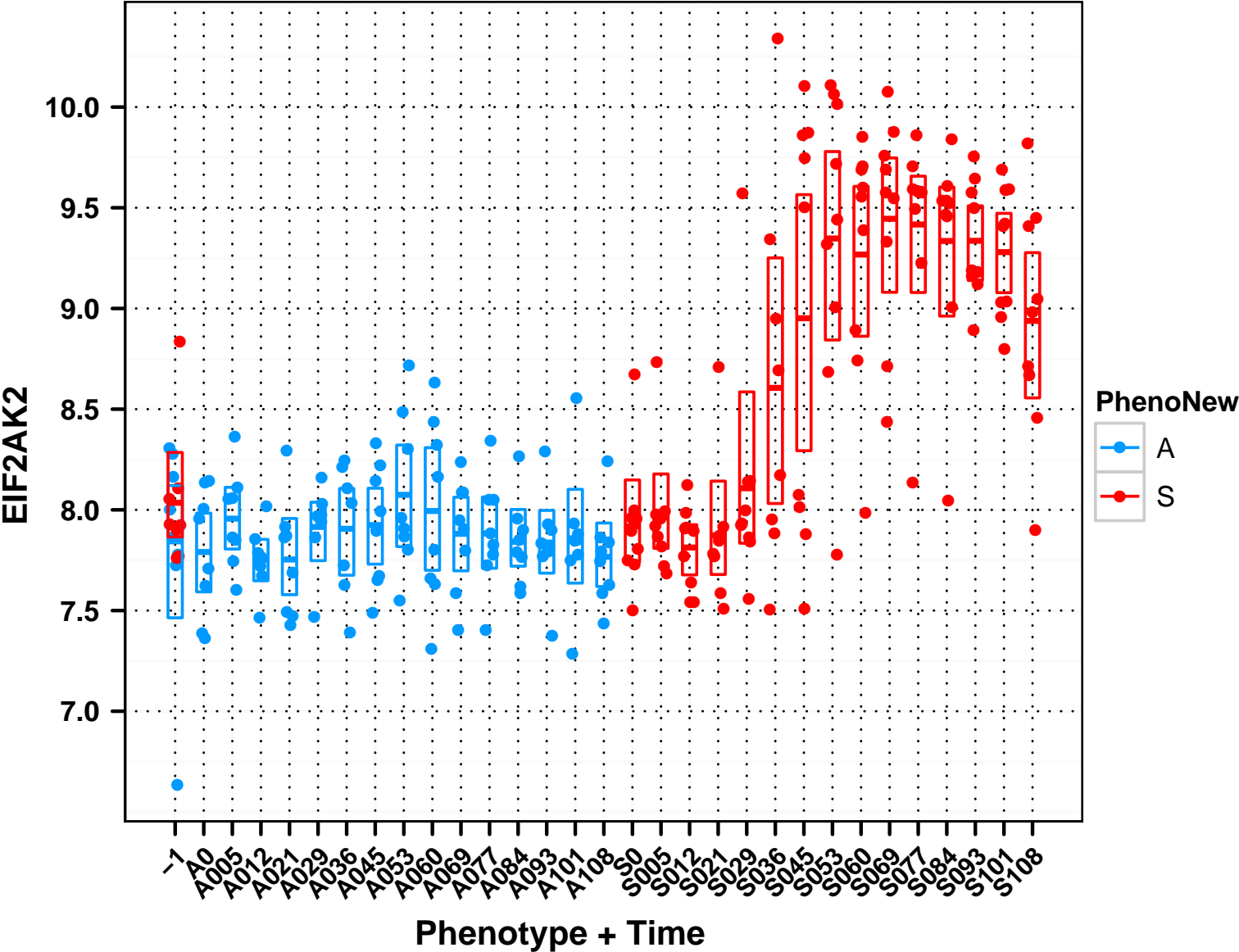

Supplement: Figure S3 — Increased temporal expression of antiviral RNA-dependent eIF-2 alpha protein kinase (EIF2AK2 or PKR) in cluster 3. The expression intensities are plotted on a log base 2 scale. (PDF) [file pgen.1002234.s003.pdf]

Fig. S4

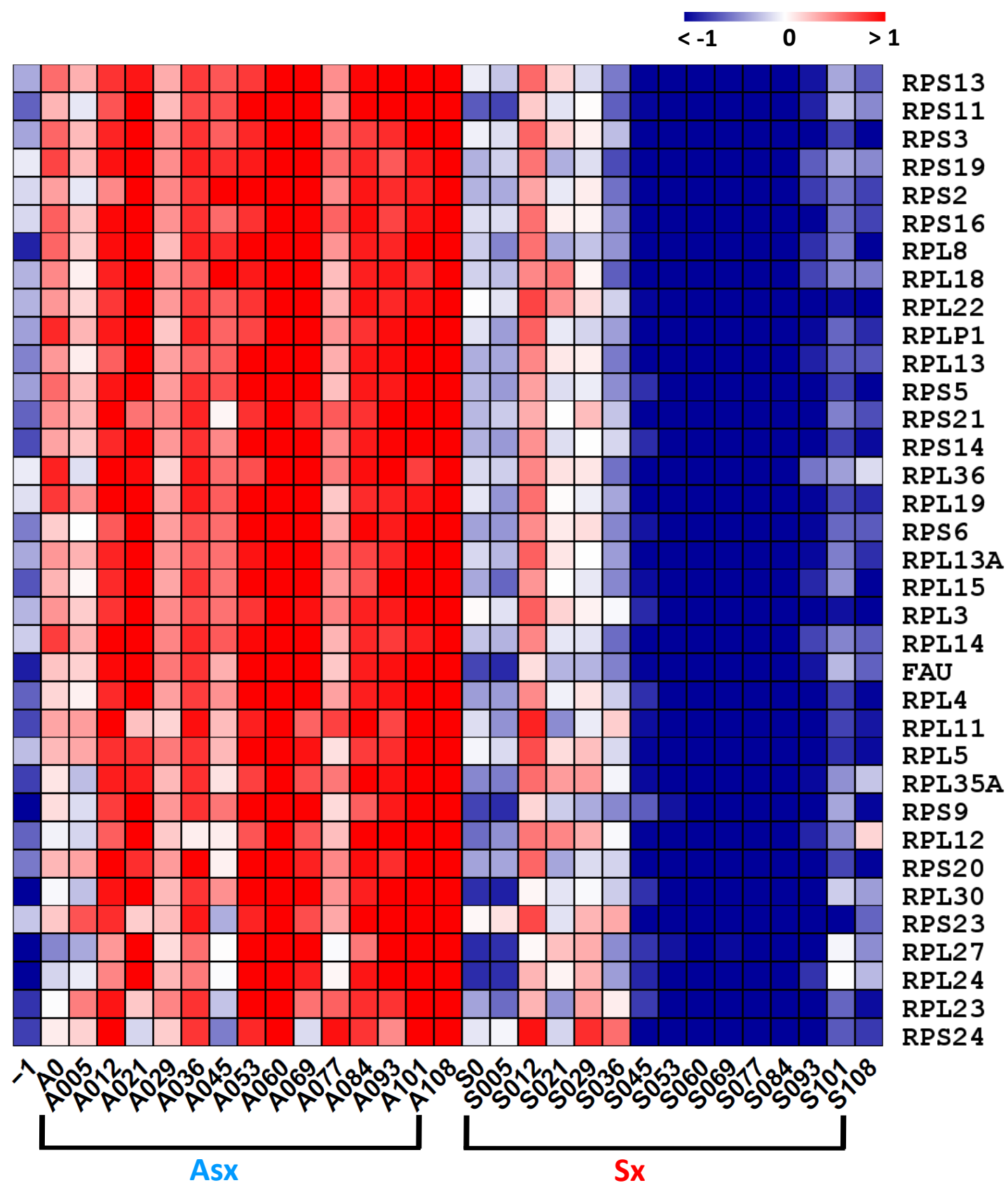

Supplement: Figure S4 — Phenotypically contrasting expression dynamics ribosomal protein synthesis-related genes (n = 35) in cluster 6. The expression intensities are averaged over subjects in Asx and Sx phenotypes and normalized to have zero mean and unit standard deviation. (PDF) [file pgen.1002234.s004.pdf]

Fig. S5

A

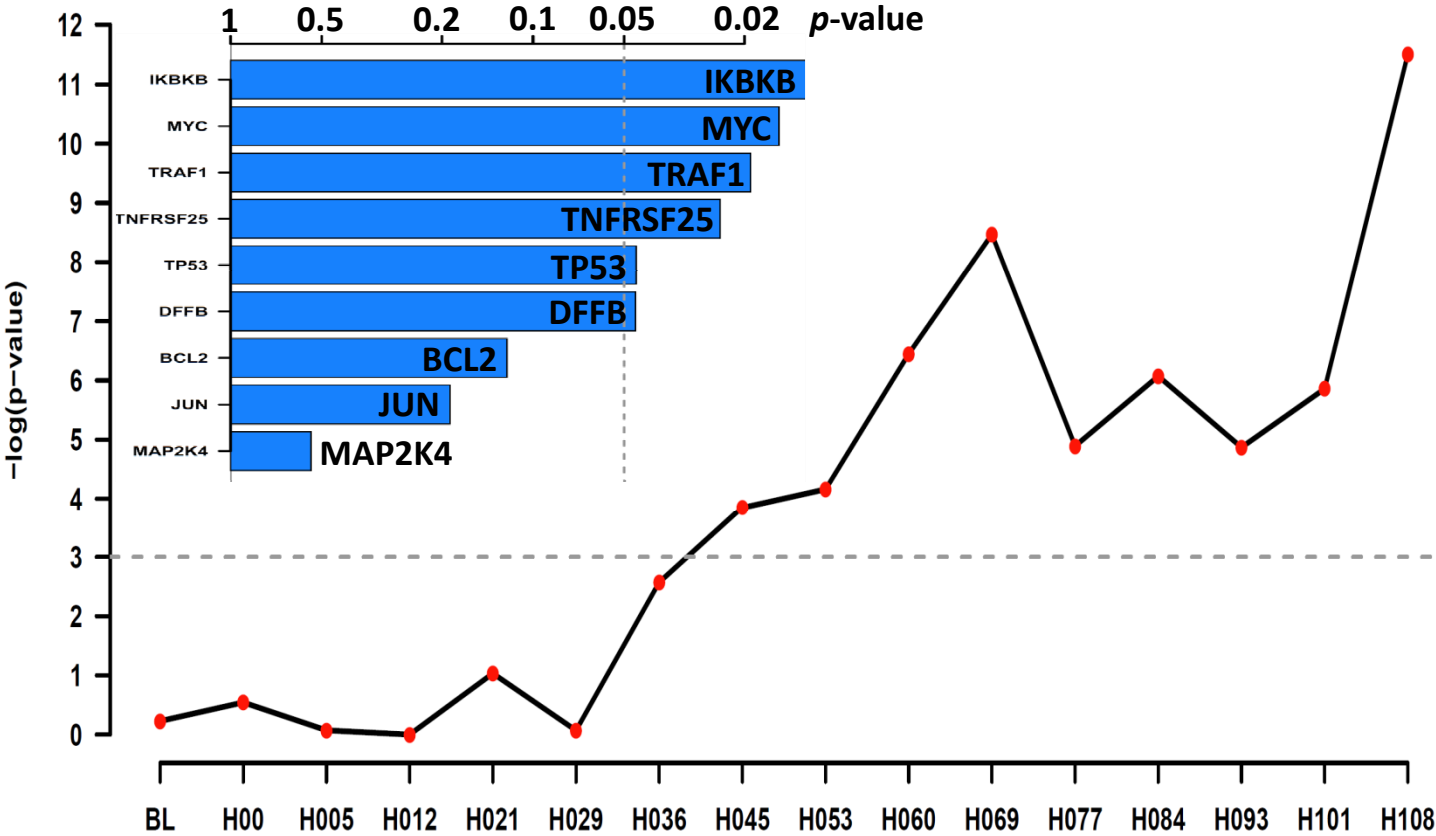

B

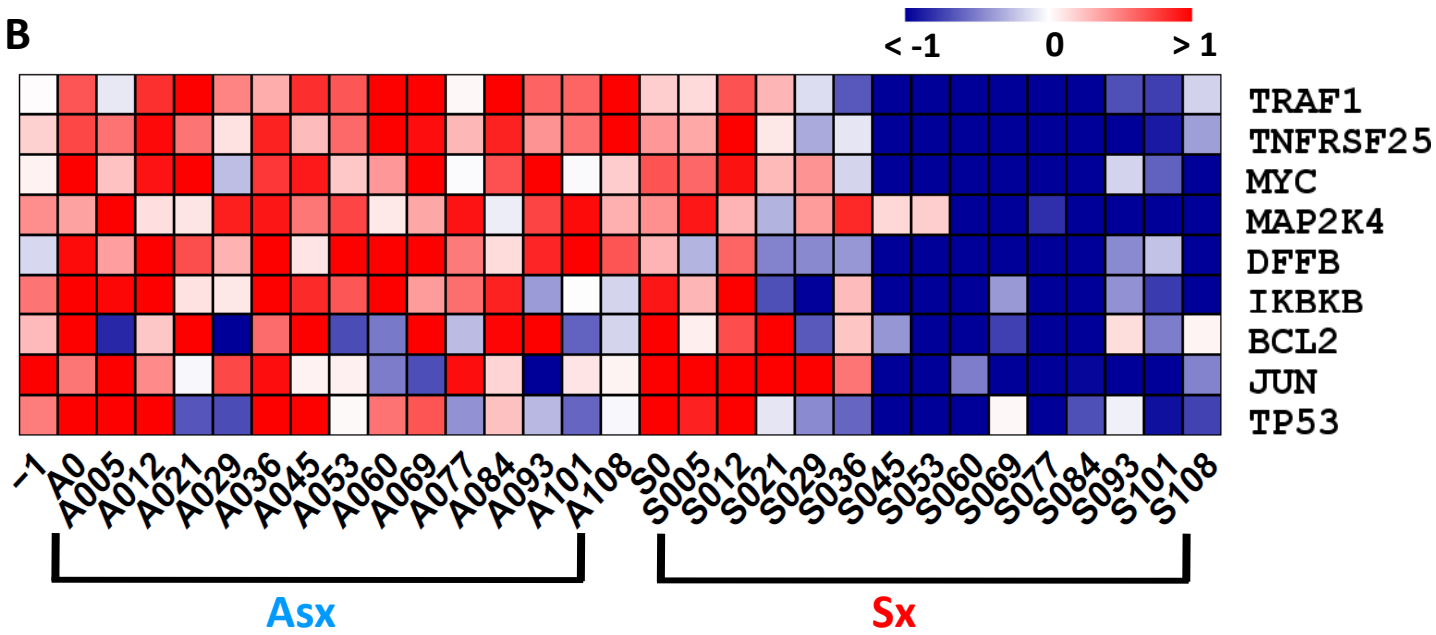

Supplement: Figure S5 — Symptomatic-specific temporal downregulation of cluster 4 genes (n = 9) that regulate programmed cell death (apoptosis). A) Significance (p-value) of association between phenotypes and the whole group of genes at all time points and at time 45 hpi (top left panel). B) Average temporal expression intensities are computed on subjects in Asx and Sx phenotypes and normalized to have zero mean and unit standard deviation. (PDF) [file pgen.1002234.s005.pdf]

Fig. S6

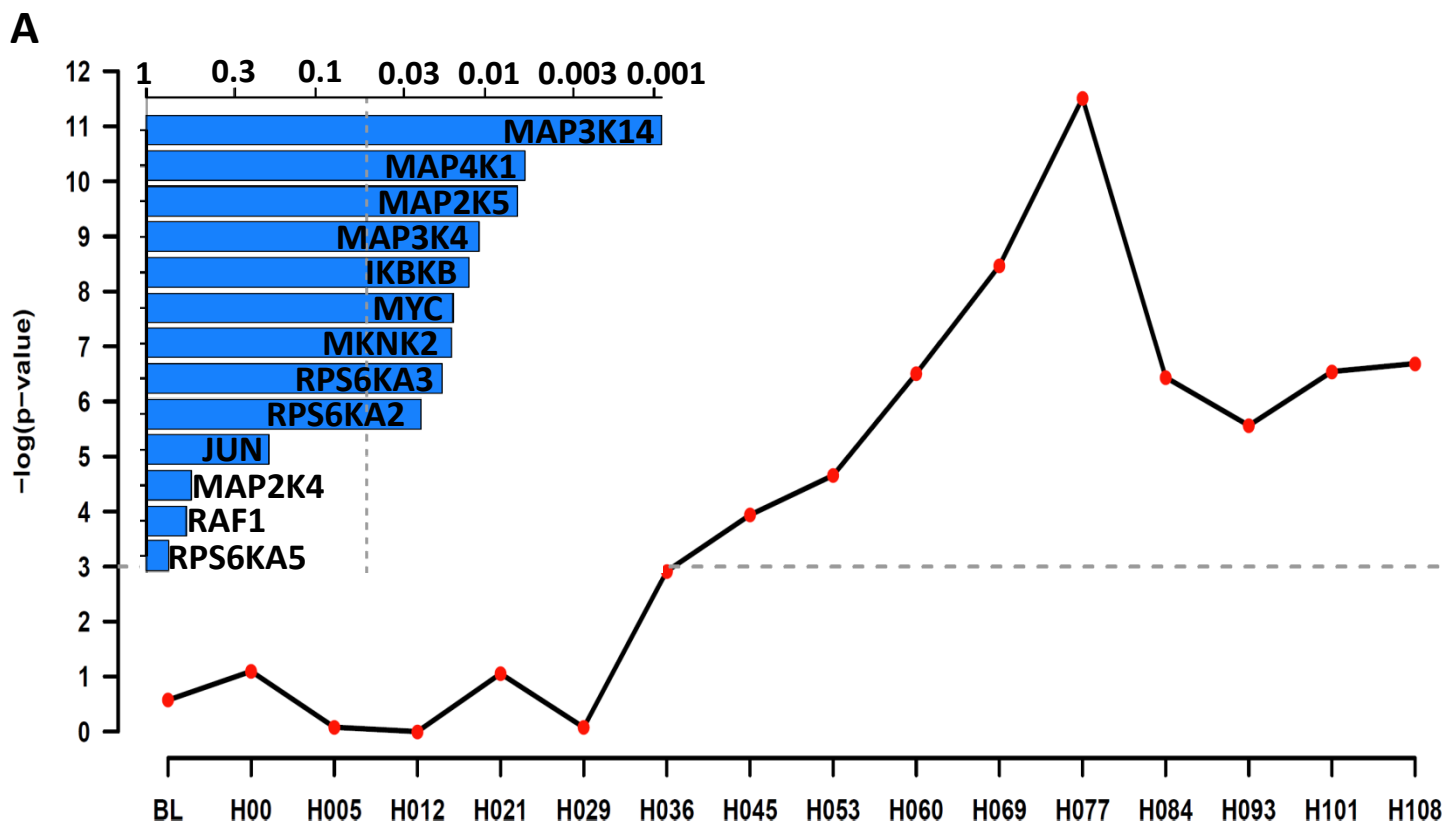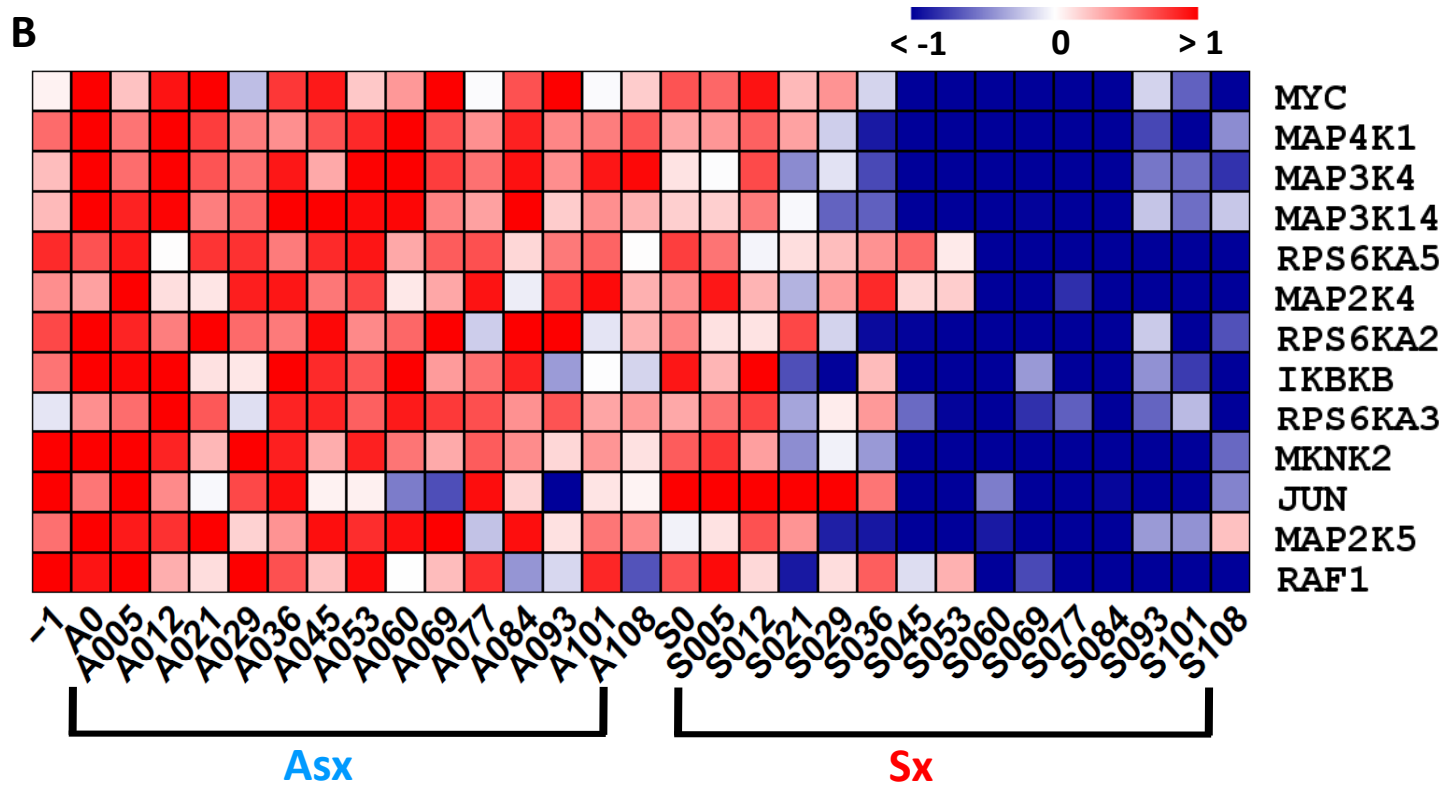

Supplement: Figure S6 — Symptomatic-specific temporal downregulation of cluster 4 genes (n = 13) that are related to mitogen-activated protein (MAP) kinase cascades. A) Significance (p-value) of association between phenotypes and the whole group of genes at all time points and at time 45 hpi (top left panel). B) Average temporal expression intensities were computed on subjects in Asx and Sx and normalized to have zero mean and unit standard deviation. (PDF) [file pgen.1002234.s006.pdf]

Fig. S7

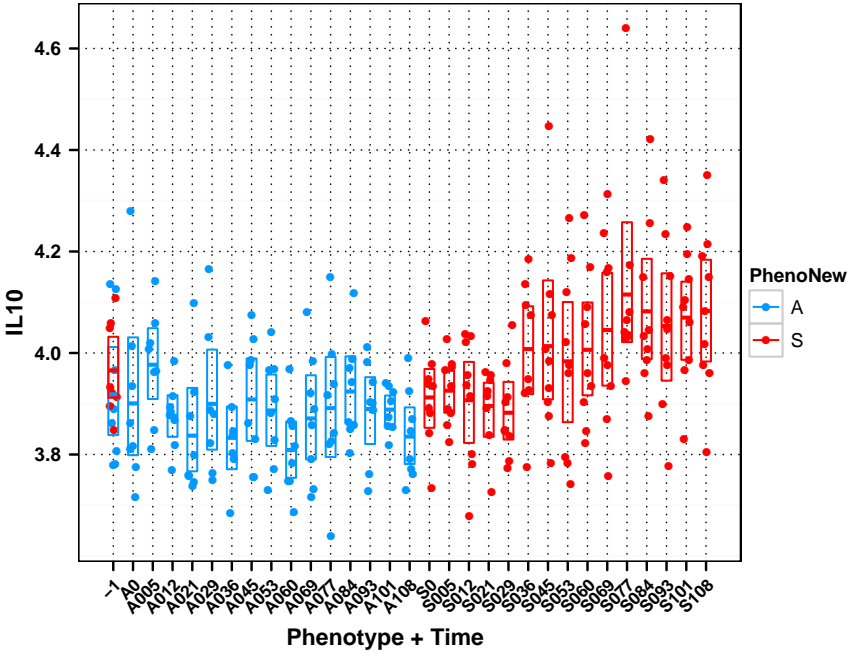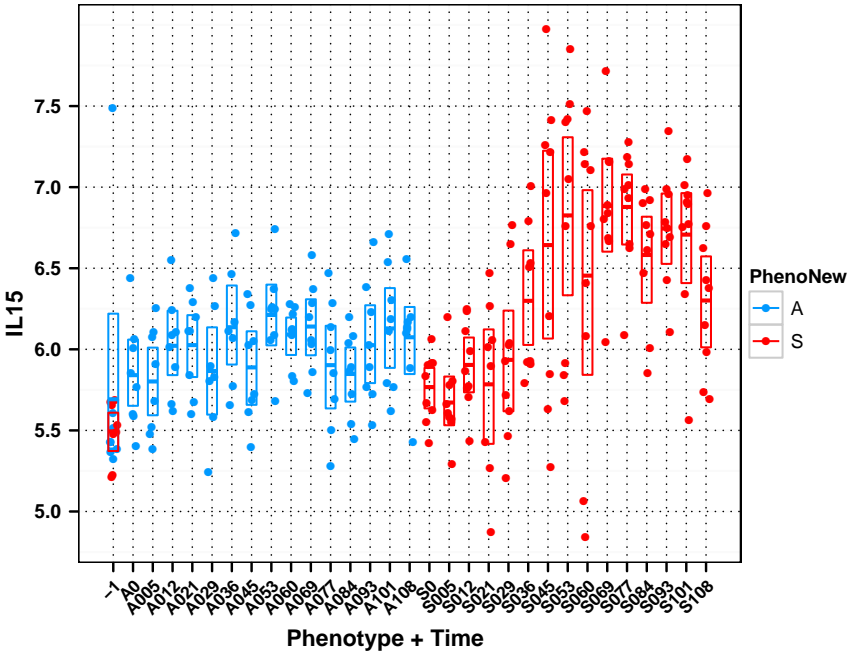

Supplement: Figure S7 — Increased temporal expression of inflammatory response regulators (cluster 3), interleukin 15 and interleukin 10. The expression intensities are plotted on a log base 2 scale. (PDF) [file pgen.1002234.s007.pdf]

Fig. S8

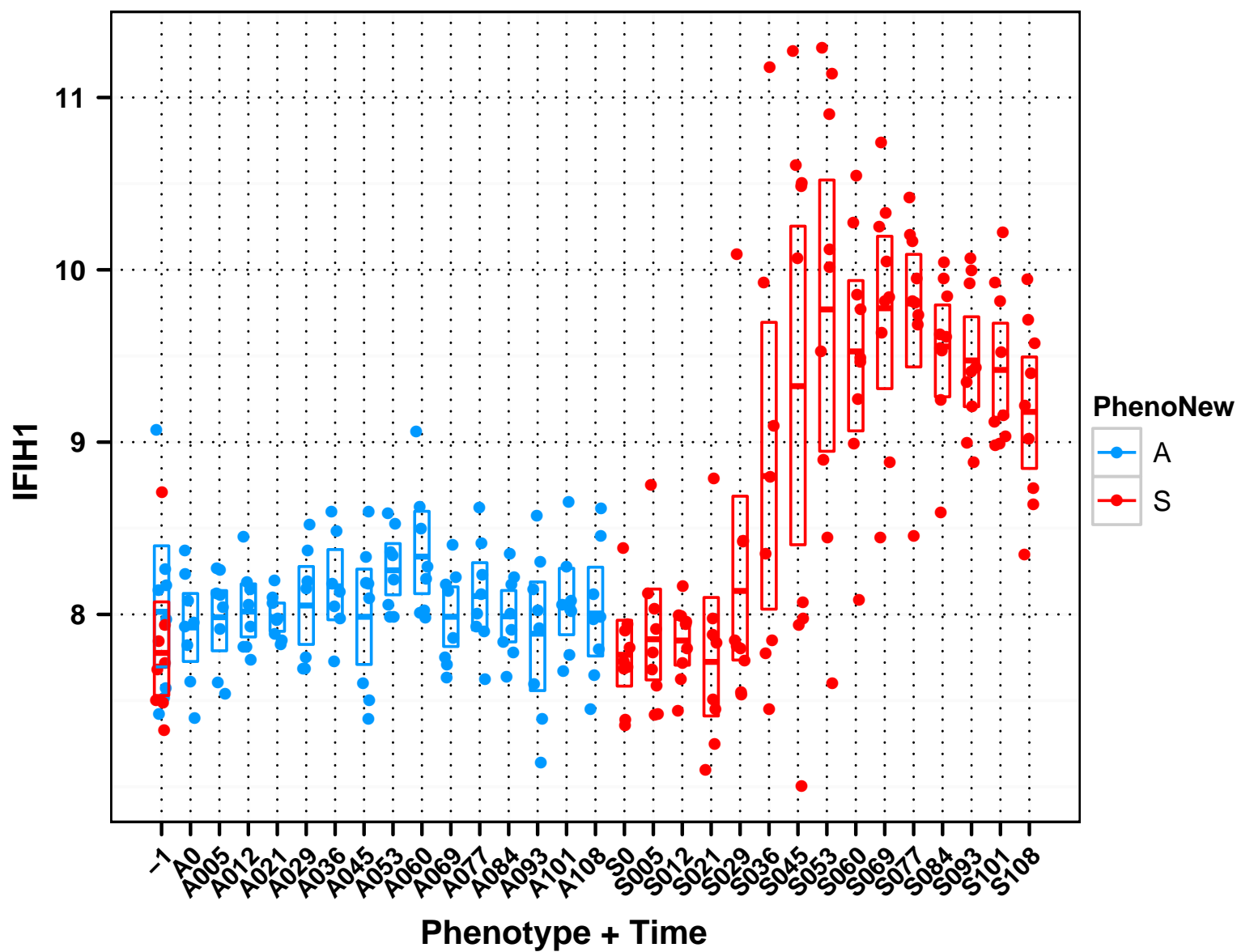

Supplement: Figure S8 — Temporal gene expression of cluster 3 gene cytoplasmic double-strand viral RNA sensor IFIH1 (interferon induced with helicase C domain 1). The expression intensities are plotted on a log base 2 scale. (PDF) [file pgen.1002234.s008.pdf]

Fig. S9

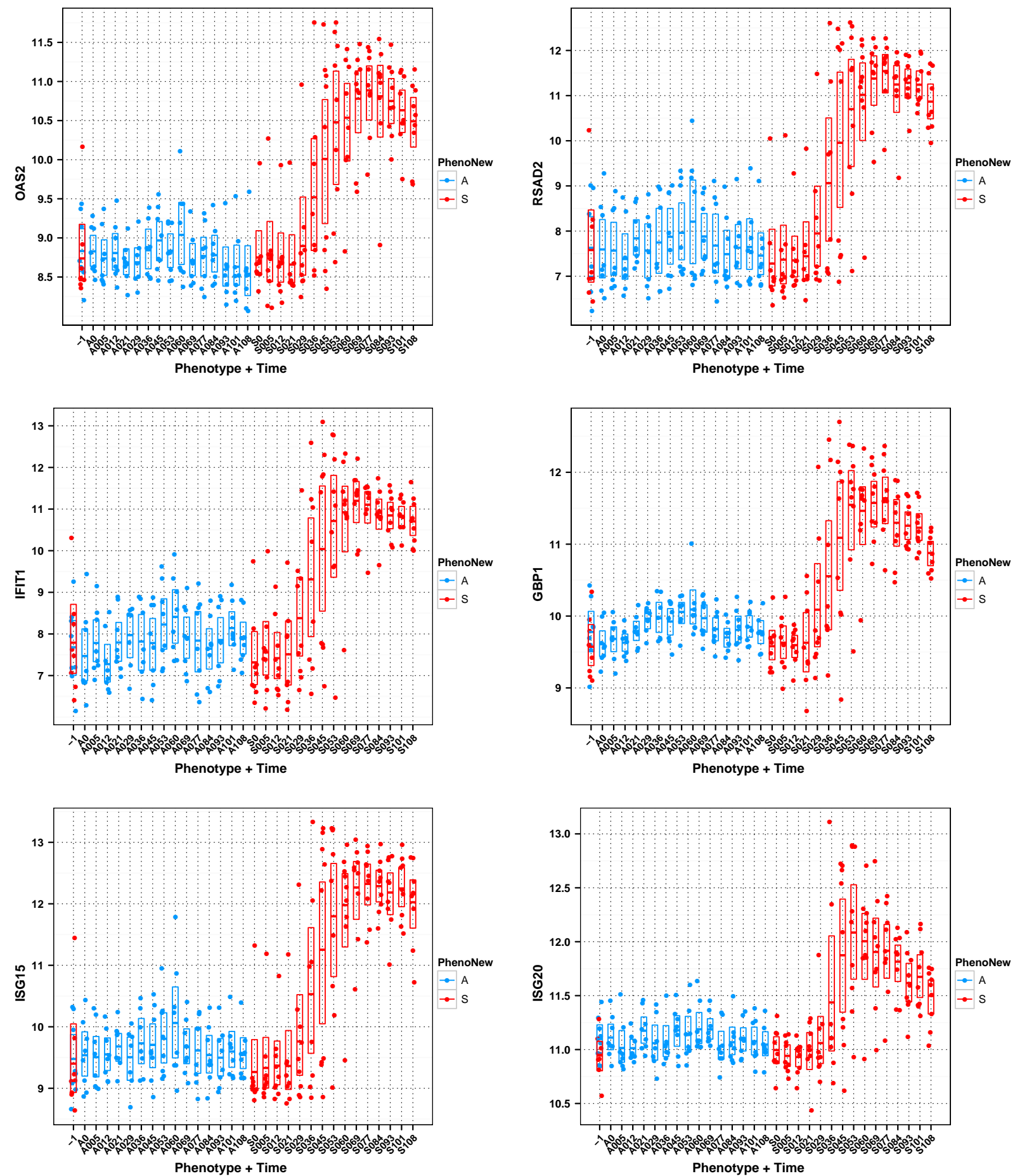

Supplement: Figure S9 — Temporal expression of interferon inducible anti-viral genes from cluster 3. The expression intensities are plotted on a log base 2 scale. (PDF) [file pgen.1002234.s009.pdf]

Fig. S10

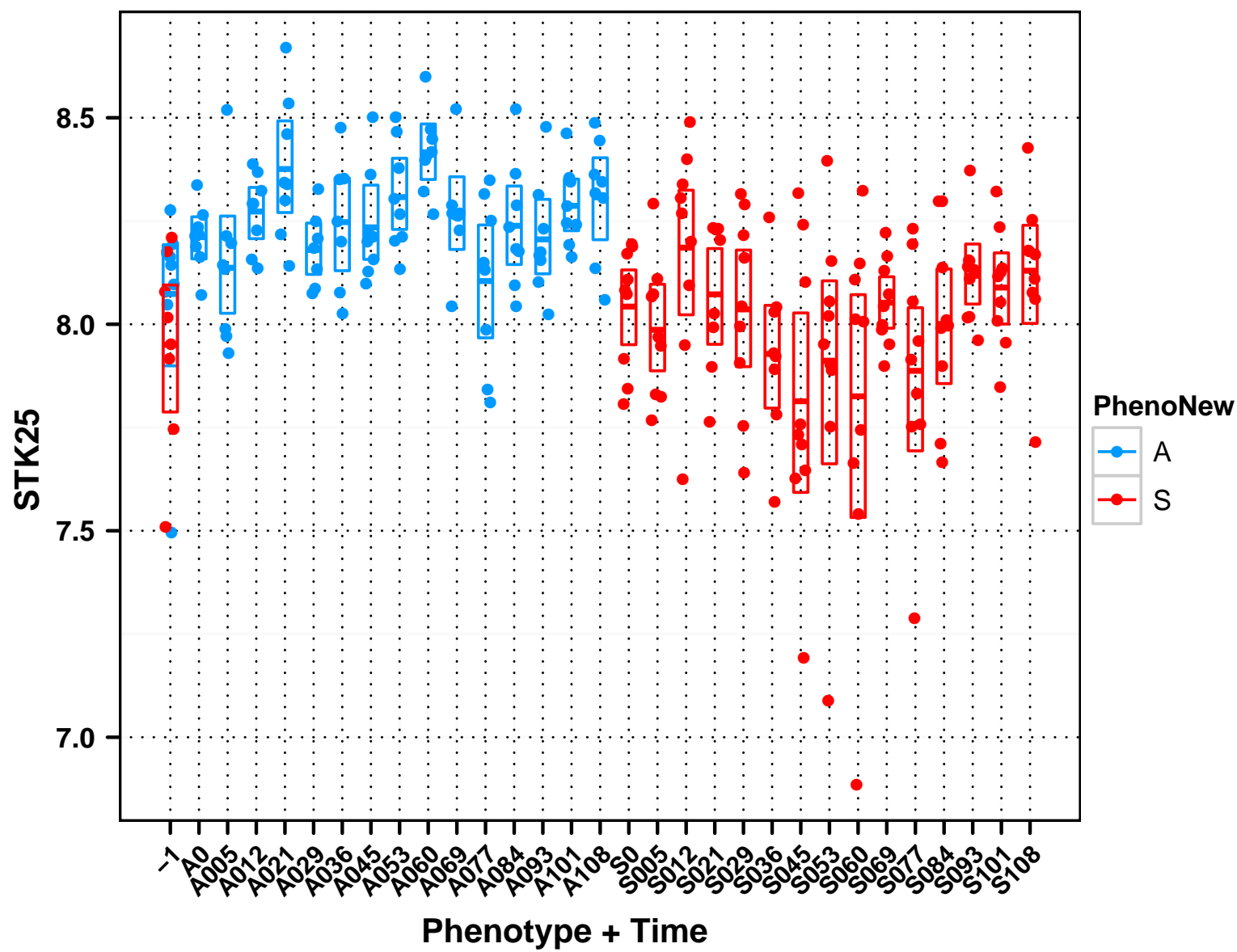

Supplement: Figure S10 — Temporal gene expression of cluster 6 gene serine/threonin kinase 25 (STK25 or SOK1). The expression intensities are plotted on a log base 2 scale. (PDF) [file pgen.1002234.s010.pdf]

**Fig. 511**

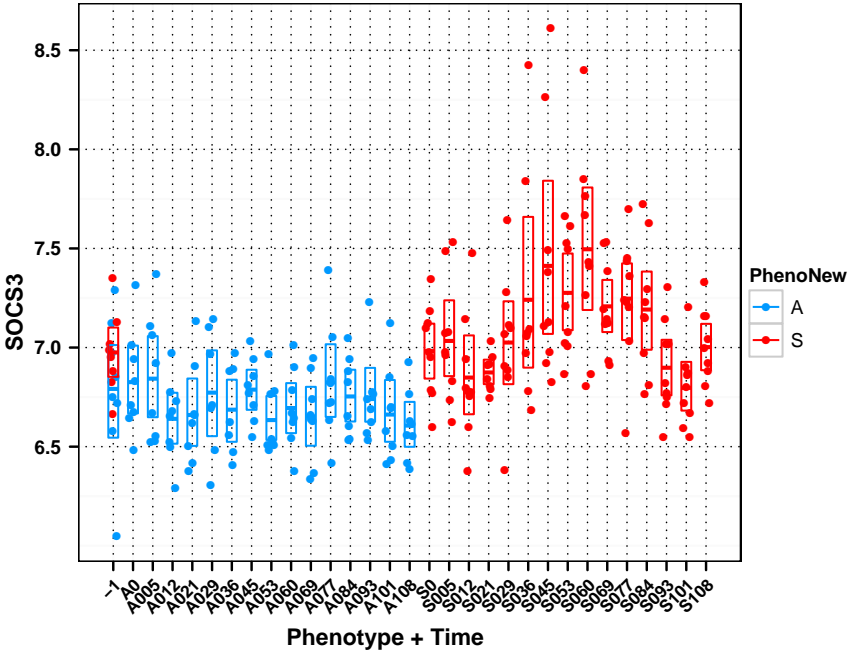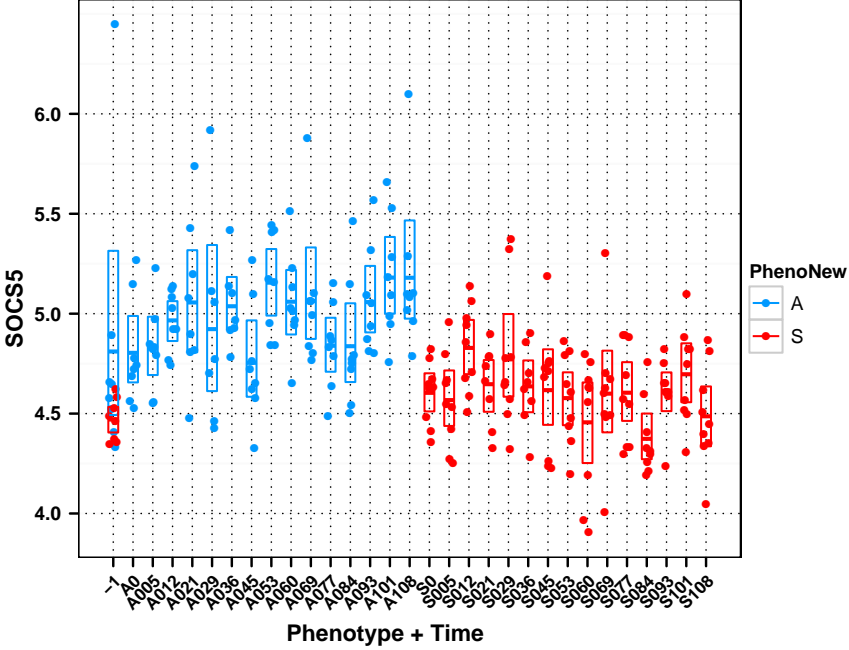

Supplement: Figure S11 — Temporal expression of genes from the family of suppressor of cytokine signaling (SOCS), including cluster 2 gene SOCS3 and cluster 6 gene SOCS5. The expression intensities are plotted on a log base 2 scale. (PDF) [file pgen.1002234.s011.pdf]

Fig. S12

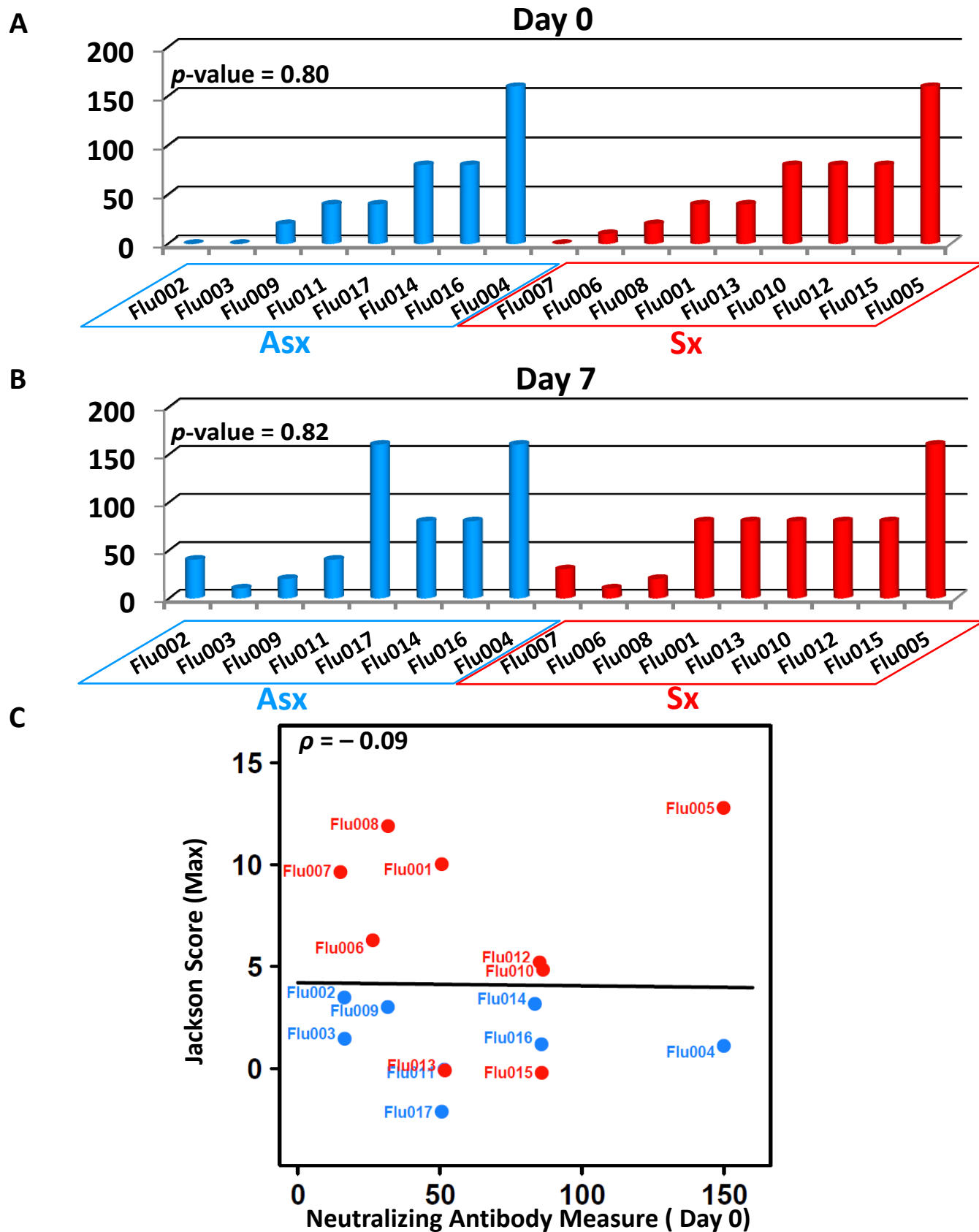

Fig. S12 (Ctd).

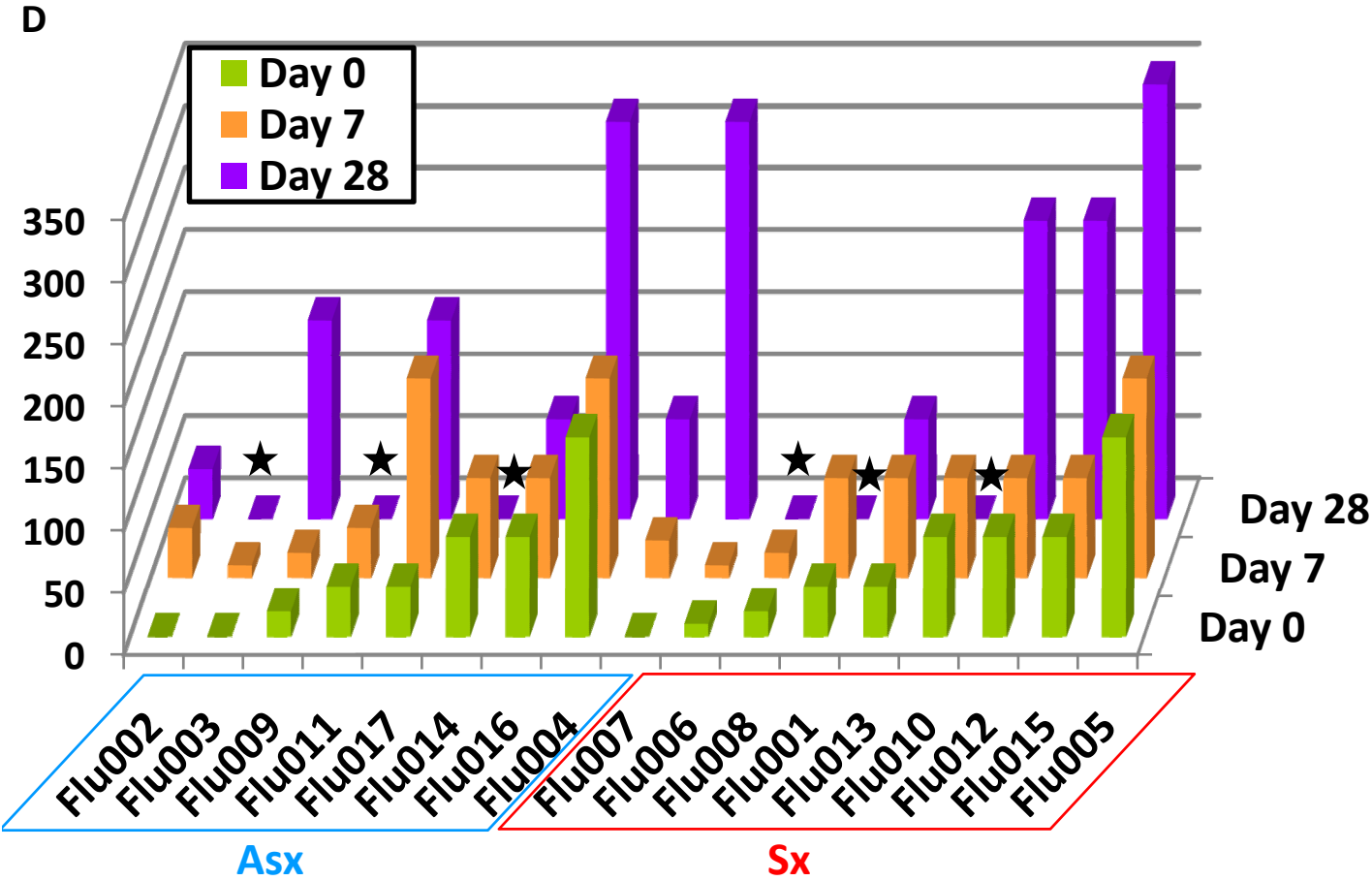

Supplement: Figure S12 — Neutralizing antibody (nAb) measure prior to inoculation shows no significant phenotypic difference and is not correlated with disease outcome. A, B) nAb of all subjects at Day 0 (A) and day 7 (B). No difference were observed between Asx and Sx on both days (non-parametric rank test). C) No evident correlation between nAb on Day 0 and maximum Jackson standardized score. A linear regression fit of score on nAb readings is shown in dark black line. Correlation test was performed using Spearman test. D) nAb increased in both Asx and Sx subjects from day 0 to day 28. ★ No sample available on day 28. (PDF) [file pgen.1002234.s012.pdf]

Fig. S13

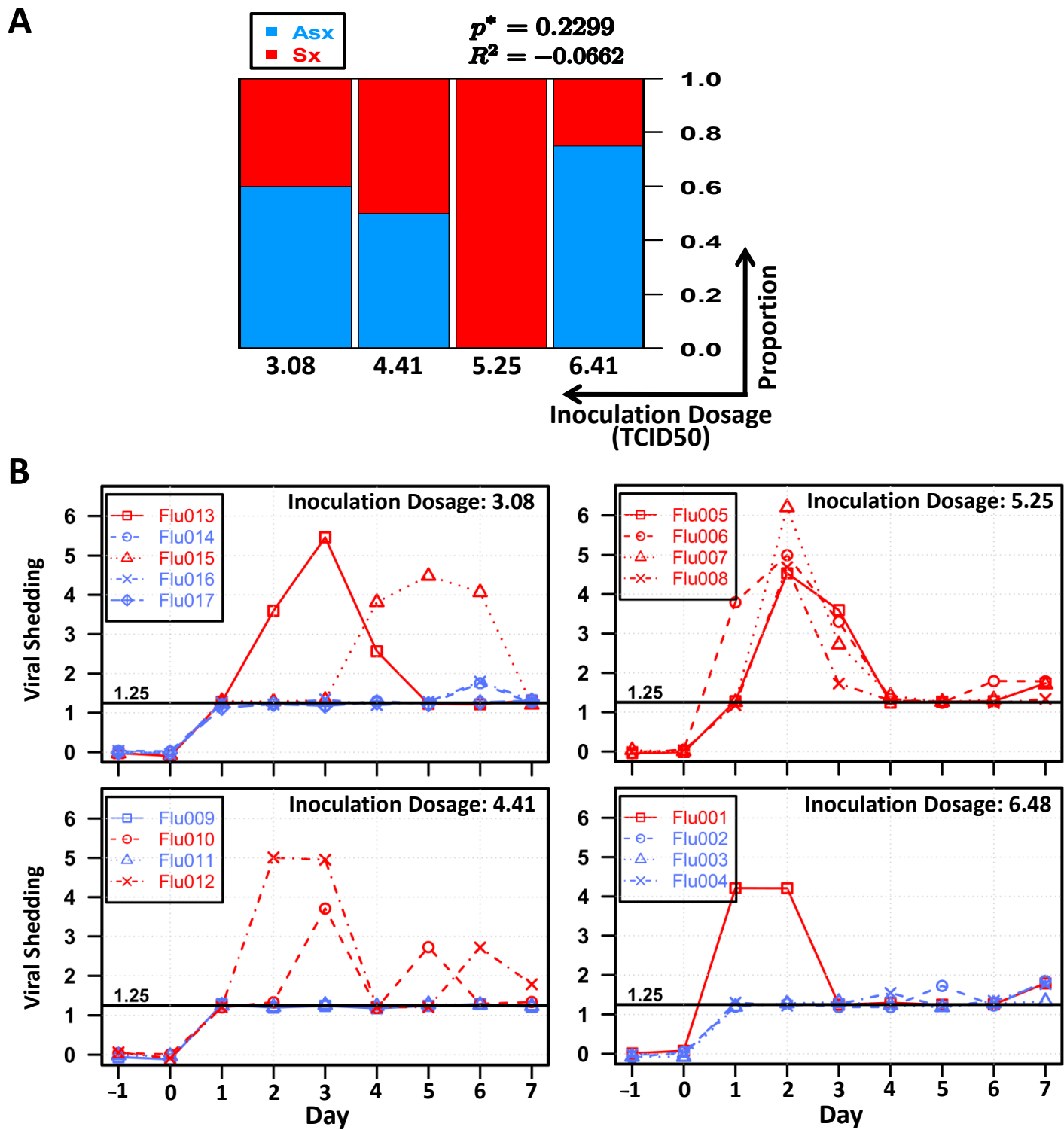

Supplement: Figure S13 — The infection outcome and viral load are independent of the dosage of viral inoculation. (A) There is no significant correlation between disease outcome and inoculation dosage (p-value = 0.2299; Fisher's exact test). Each bar represents a randomized group of four to five subjects receiving a varying dose of Influenza A virus inoculation on day 0 (Supplementary Materials). Within each group, subjects are divided into clinically determined symptomatic (red) and asymptomatic (blue) subgroups. (B) Viral shedding pattern (Table S2A) does not differ across inoculation dosage groups. All nine symptomatic and four asymptomatic subjects showed shedding ≥1.25. Two asymptomatic shedders (#14 and #16) are in lowest dosage group and the other two (#2 and #4) are in the highest dosage group. The amount of viral shedding are determined from nasal wash obtained daily (Supplementary Methods). Shedding values <1.25 are set to 1.25 in the plot. (PDF) [file pgen.1002234.s013.pdf]

Fig S14

A

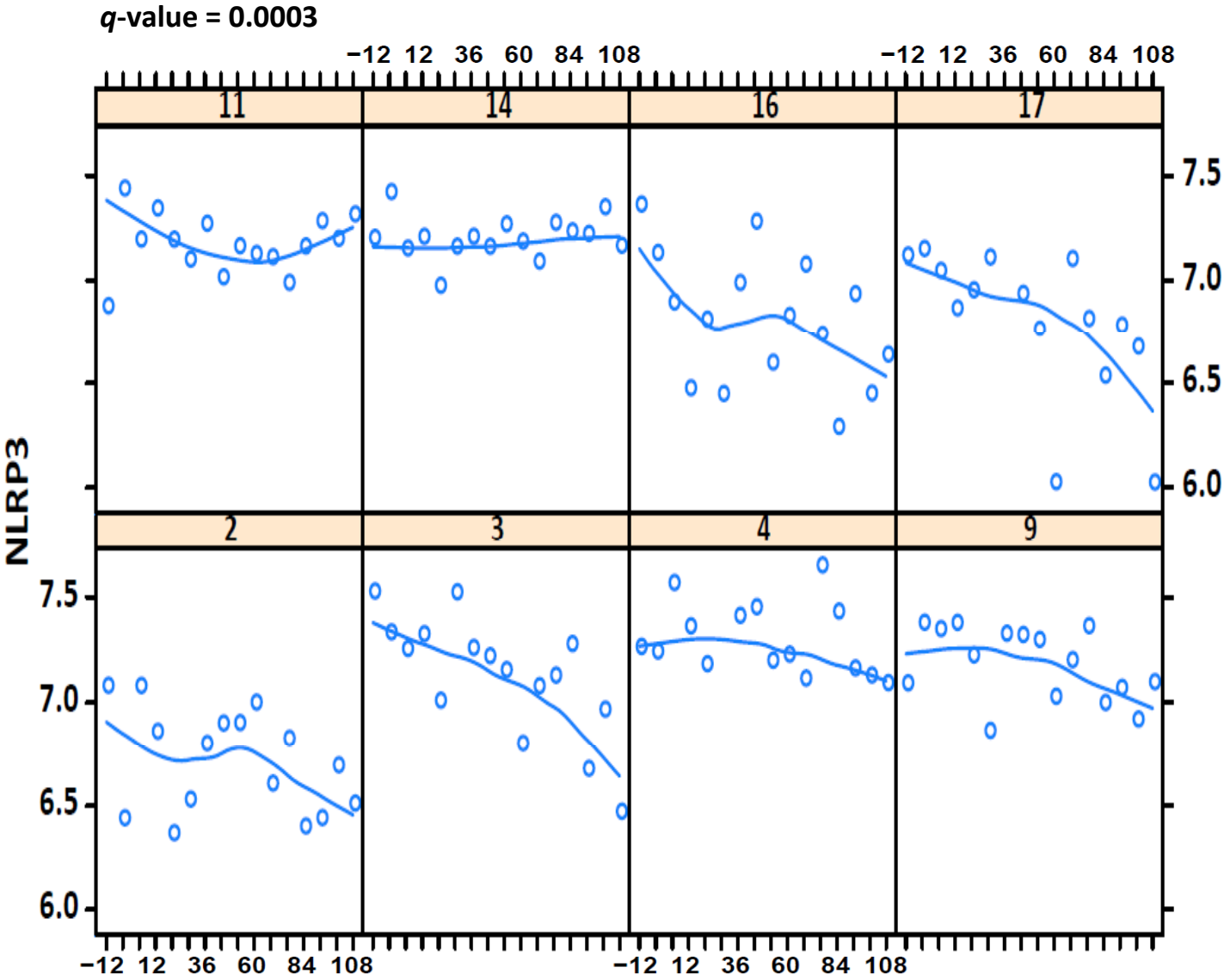

Supplement: Figure S14 — Asymptomatic subjects demonstrated non-passive transcriptional response program. As an example, we show a significant temporal expression decrease of the inflammasome related gene NLRP3 in eight individual asymptomatic subjects. Each subpanel depicts the temporal expression of one individual asymptomatic subject. The y-axis is the log base 2 signal intensity of NLRP3 and the x-axis is the time from −12 hpi to 108 hpi (hour post inoculation). A polynomial fitting of expression values (solid line) was fitted using LOESS model and significance of temporal trend was assessed with EDGE. Subjects #3 and #17 never showed detectable amount of virus (<1.25) in their nasal wash (Table S2). (PDF) [file pgen.1002234.s014.pdf]

Fig. S15

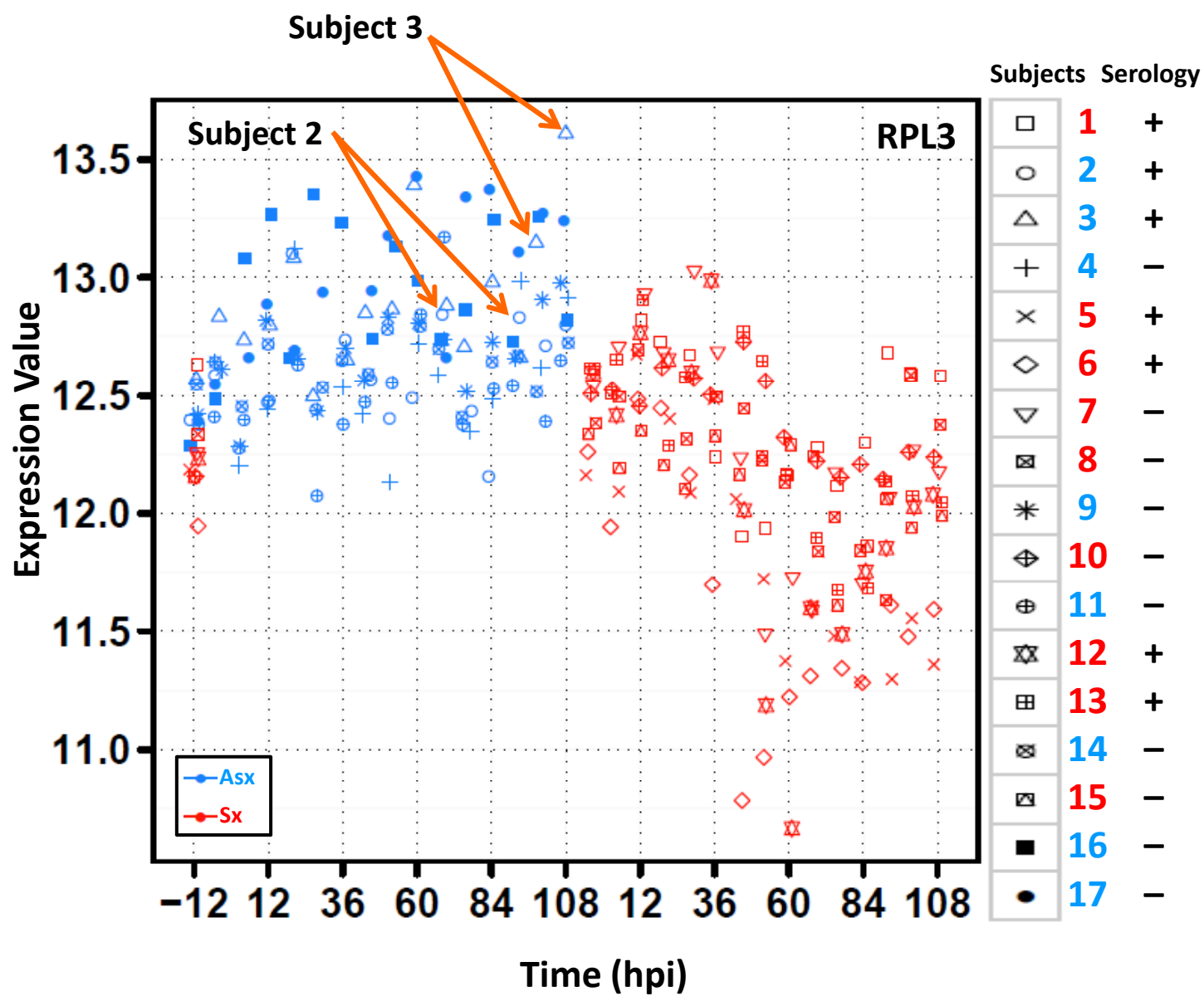

Supplement: Figure S15 — Serological conversion versus clinical symptom outcome and gene expression. The RPL3 gene expression trajectories for Asx (blue) and Sx (red) are representative of SOM cluster 6. Legend at right gives the character encoding of each subject along with their disease outcome (‘blue’ Asx and ‘red’ Sx) and their serologic conversion outcome (‘+’ converted and ‘−’ not converted). There is no significant relation between disease outcome and serological conversion (p-value of 0.27 according to likelihood ratio test of dependency between these two outcomes). The two seroconverted asymptomatic individuals (subject #2 and #3) are called out by orange arrows in the gene expression trajectory plot. The RPL3 expression profiles of these two subjects are not significantly different from those of the other asymptomatic hosts. (PDF) [file pgen.1002234.s015.pdf]

Fig. S16

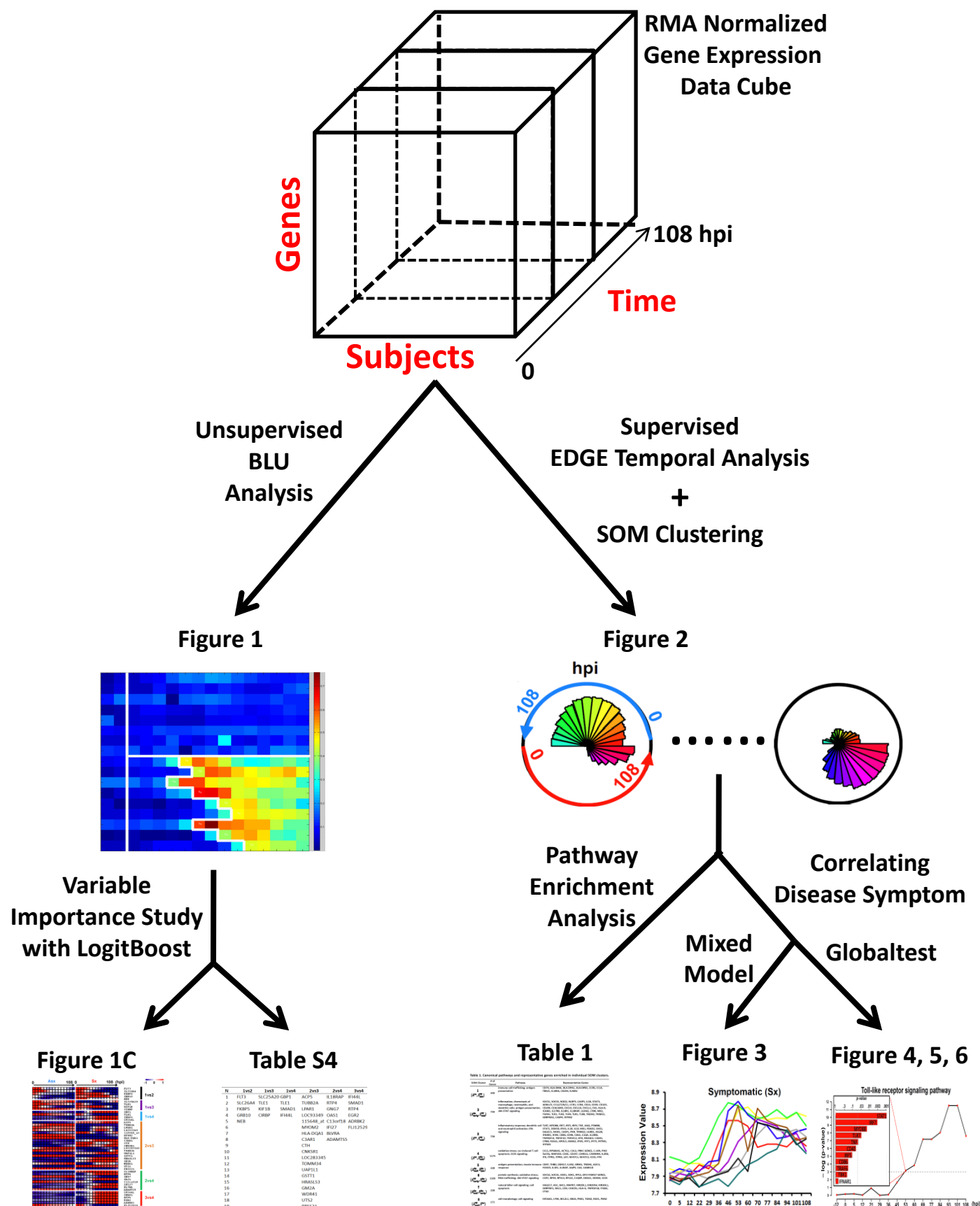

Supplement: Figure S16 — Schematic outline of analysis pipeline. Unsupervised: no clinical phenotype information was used. Supervised: clinical phenotype was incorporated in analysis. (PDF) [file pgen.1002234.s016.pdf]

Fig. S17

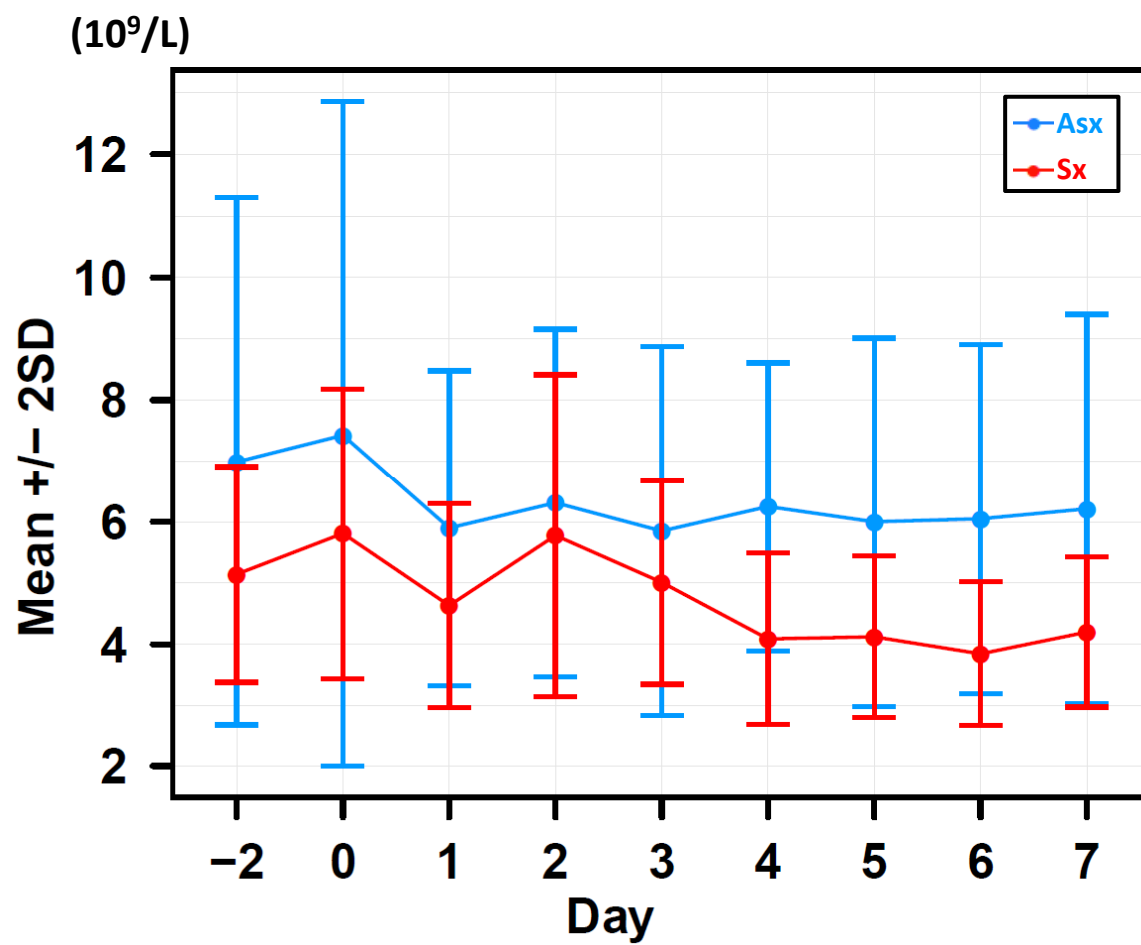

Supplement: Figure S17 — Daily white blood cell counts show mild change (less than 80%) from baseline in Asx and Sx phenotypes. (PDF) [file pgen.1002234.s017.pdf]

Fig. S18

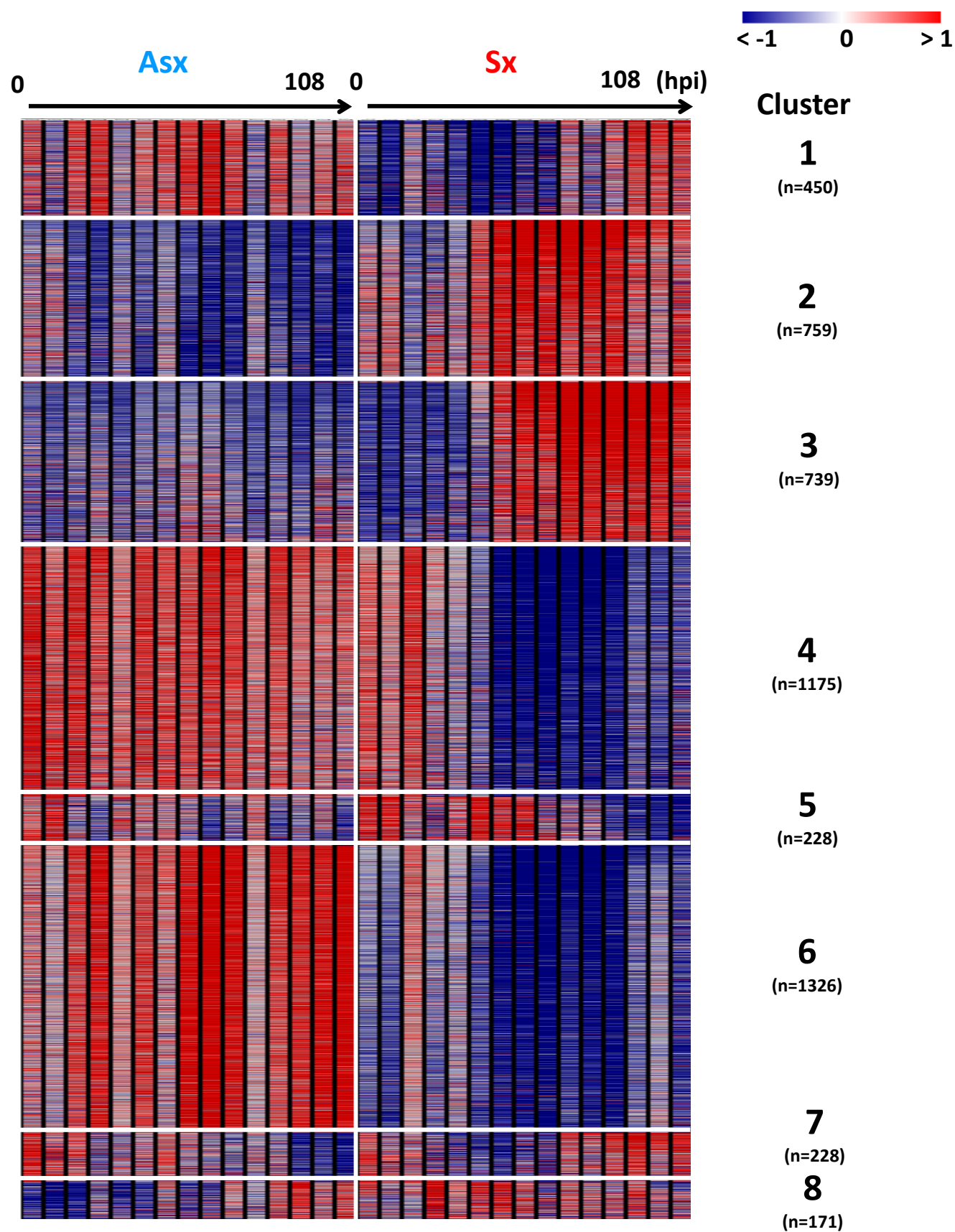

Supplement: Figure S18 — Expression heatmap of genes that are significantly differentially expressed between Asx and Sx. Genes are identified using EDGE (q-value<0.01) and clustered with SOM. The average expression are computed and normalized for each gene to have zero mean and unit standard deviation. Within a cluster, genes are shown in decreasing order of significance level. (PDF) [file pgen.1002234.s018.pdf]
