# Supplementary material for: Temporal Dynamics of Host Molecular Responses Differentiate Symptomatic and Asymptomatic Influenza A Infection
Source: PLoS Genet. 2011 Aug 25;7(8):e1002234. doi: 10.1371/journal.pgen.1002234 (PMC3161909; doi:10.1371/journal.pgen.1002234)
Supplement: Table S1 — (A) Subject Demographic and Clinical Characteristics of Viral Challenge Cohort. (B) Detailed subject demographics. (PDF) [file pgen.1002234.s019.pdf]

Table S1

A

CHARACTERISTICs	ALL SUBJECTs	ASYMPTOMATIC (Asx)	SYMPTOMATIC (Sx)
No. of subjects	17	8	9
Female sex (%)	8 (47%)	3 (38%)	5 (56%)
Age (min, max)	27 (22, 41)	28 (22, 41)	27 (22, 35)
White race (%)	14 (82%)	7 (88%)	7 (78%)
Average symptom score (min, max)	2 (0, 15)	(0, 1)	4 (0, 15)
Average peak symptom (min, max)	8 (0, 15)	0 (0, 1)	12 (8, 16)

B

Class	Unique ID	Gender	Age (years)	Race/Ethnicity
**Asx**	**flu011**	Female	25	Caucasian/White
	**flu002**	Male	28	Caucasian/White
	**flu003**	Male	24	Caucasian/White
	**flu004**	Female	23	Caucasian/White
	**flu009**	Male	24	Caucasian/White
	**flu014**	Female	22	Caucasian/White
	**flu016**	Male	41	Caucasian/White
	**flu017**	Male	33	Indian (Indian Subcontinent)
**Sx**	**flu013**	Female	29	Caucasian/White
	**flu015**	Male	26	Caucasian/White
	**flu001**	Female	29	Black/Africa Origin
	**flu005**	Female	25	Caucasian/White
	**flu006**	Female	28	Mixed Ethnicity
	**flu007**	Male	35	Caucasian/White
	**flu008**	Male	25	Caucasian/White
	**flu010**	Female	22	Caucasian/White
	**flu012**	Male	27	Caucasian/White
