# Supplementary material for: Temporal Dynamics of Host Molecular Responses Differentiate Symptomatic and Asymptomatic Influenza A Infection
Source: PLoS Genet. 2011 Aug 25;7(8):e1002234. doi: 10.1371/journal.pgen.1002234 (PMC3161909; doi:10.1371/journal.pgen.1002234)
Supplement: Table S2 — Viral shedding and serological testing data for all human volunteers (n = 17) challenged with Influenza H3N2 viruses. A) Measure of viral titre isolated from nasal wash over a total of 9 days. B) Serological data on pre-screening, −24 hpi, and +28 days. (PDF) [file pgen.1002234.s020.pdf]

Table S2

A

Pheno	Unique ID	Day -2	Day -1	Day 0	Day 1	Day 2	Day 3	Day 4	Day 5	Day 6	Day 7
	Virus Isolation (from nasal wash)										
Asx	flu002	none detected	none detected	none detected	<1.25	<1.25	<1.25	<1.25	1.75	<1.25	1.75
Asx	flu003	none detected	none detected	none detected	<1.25	<1.25	<1.25	<1.25	<1.25	<1.25	<1.25
Asx	flu004	none detected	none detected	none detected	<1.25	<1.25	<1.25	1.5	<1.25	<1.25	1.75
Asx	flu009	none detected	none detected	none detected	<1.25	<1.25	<1.25	<1.25	<1.25	<1.25	<1.25
Asx	flu 011	none detected	none detected	none detected	<1.25	<1.25	<1.25	<1.25	<1.25	<1.25	<1.25
Asx	flu014	none detected	none detected	none detected	<1.25	<1.25	<1.25	<1.25	<1.25	1.75	<1.25
Asx	flu016	none detected	none detected	none detected	<1.25	<1.25	<1.25	<1.25	<1.25	1.75	<1.25
Asx	flu017	none detected	none detected	none detected	<1.25	<1.25	<1.25	<1.25	<1.25	<1.25	<1.25
Sx	flu001	none detected	none detected	none detected	4.25	4.25	<1.25	<1.25	<1.25	<1.25	1.75
Sx	flu005	none detected	none detected	none detected	<1.25	4.5	3.5	<1.25	<1.25	<1.25	1.75
Sx	flu006	none detected	none detected	none detected	3.75	5	3.25	<1.25	<1.25	1.75	1.75
Sx	flu007	none detected	none detected	none detected	<1.25	6.25	2.75	<1.5	<1.25	<1.25	1.75
Sx	flu008	none detected	none detected	none detected	<1.25	4.75	1.75	<1.25	<1.25	<1.25	<1.25
Sx	flu010	none detected	none detected	none detected	<1.25	<1.25	3.75	<1.25	2.75	<1.25	<1.25
Sx	flu012	none detected	none detected	none detected	<1.25	5.01	5.01	<1.25	<1.25	2.75	1.75
Sx	flu013	none detected	none detected	none detected	<1.25	3.51	5.5	2.5	<1.25	<1.25	<1.25
Sx	flu015	none detected	none detected	none detected	<1.25	<1.25	<1.25	3.75	4.5	4	<1.25

B

Pheno	Unique ID	Pre-Screening visit	Day -1	Day 28 (convalescent)	Seroconversion
	Serology				
Asx	flu002	No detectable antibody	No detectable antibody	40	Yes
Asx	flu003	No detectable antibody	No detectable antibody	160	Yes
Asx	flu004	No detectable antibody	No detectable antibody	No detectable antibody	No
Asx	flu009	No detectable antibody	No detectable antibody	No detectable antibody	No
Asx	flu 011	No detectable antibody	No detectable antibody	Did not attend Day +28 visit	No
Asx	flu014	No detectable antibody	No detectable antibody	No detecable antibody	No
Asx	flu016	No detectable antibody	No detectable antibody	<20	No
Asx	flu017	No detectable antibody	No detectable antibody	No detectable antibody	No
Sx	flu001	No detectable antibody	No detectable antibody	320	Yes
Sx	flu005	No detectable antibody	No detectable antibody	320	Yes
Sx	flu006	No detectable antibody	No detectable antibody	57	Yes
Sx	flu007	No detectable antibody	No detectable antibody	<20	No
Sx	flu008	No detectable antibody	No detectable antibody	Did not attend Day +28 visit	No
Sx	flu010	No detectable antibody	No detectable antibody	Did not attend day +28 visit	No
Sx	flu012	No detectable antibody	No detectable antibody	80	Yes
Sx	flu013	No detectable antibody	No detectable antibody	40	Yes
Sx	flu015	No detectable antibody	No detectable antibody	20	No
