# Supplementary material for: Temporal Dynamics of Host Molecular Responses Differentiate Symptomatic and Asymptomatic Influenza A Infection
Source: PLoS Genet. 2011 Aug 25;7(8):e1002234. doi: 10.1371/journal.pgen.1002234 (PMC3161909; doi:10.1371/journal.pgen.1002234)
Supplement: Table S3 — Significance of monotonic trend of gene expression in SOM clusters. For the genes in each SOM cluster (Figure 1), we implemented the Jonkheere-Terpstra (JT) test (Text S1) of significance on Asx and Sx subjects, respectively, to test for monotonic increase or decrease of gene expression over time. Columns 2 and 3 show p-values associated with the null hypothesis that genes in the cluster have no monotonic trend. Red colored entries indicate clusters having highly significant monotonic expression profiles for a particular phenotype. (PDF) [file pgen.1002234.s021.pdf]

Table S3

SOM Cluster	Asymptomatic (ASX)	Symptomatic (SX)
FLU1	*0.2964*	***< 0.0001***
FLU2	***< 0.0001***	***< 0.0001***
FLU3	*0.3924*	***< 0.0001***
FLU4	***< 0.0001***	***< 0.0001***
FLU5	***0.0002***	***< 0.0001***
FLU6	***0.0002***	***< 0.0001***
FLU7	***< 0.0001***	*0.1594*
FLU8	***< 0.0001***	*0.8264*
