# Supplementary material for: Temporal Dynamics of Host Molecular Responses Differentiate Symptomatic and Asymptomatic Influenza A Infection
Source: PLoS Genet. 2011 Aug 25;7(8):e1002234. doi: 10.1371/journal.pgen.1002234 (PMC3161909; doi:10.1371/journal.pgen.1002234)
Supplement: Table S4 — Discriminatory genes selected by each logistic boosting model. Genes are listed in decreasing order based on their discriminatory power in each model. (PDF) [file pgen.1002234.s022.pdf]

Table S4

N	1vs2	1vs3	1vs4	2vs3	2vs4	3vs4
1	FLT3	SLC25A20	GBP1	ACP5	IL18RAP	IFI44L
2	SLC26A4	TLE1	TLE1	TUBB2A	RTP4	SMAD1
3	FKBP5	KIF1B	SMAD1	LPAR1	GNG7	RTP4
4	GRB10	CIRBP	IFI44L	LOC93349	OAS1	EGR2
5	NEB			115648_at	C13orf18	ADRBK2
6				MYOM2	IFI27	FLJ12529
7				HLA-DQA1	BLVRA	
8				C3AR1	ADAMTS5	
9				CTH		
10				CNKSR1		
11				LOC283345		
12				TOMM34		
13				UAP1L1		
14				GSTT1		
15				HRASLS3		
16				GM2A		
17				WDR41		
18				UTS2		
19				PRSS21		
