# Supplementary material for: Temporal Dynamics of Host Molecular Responses Differentiate Symptomatic and Asymptomatic Influenza A Infection
Source: PLoS Genet. 2011 Aug 25;7(8):e1002234. doi: 10.1371/journal.pgen.1002234 (PMC3161909; doi:10.1371/journal.pgen.1002234)
Supplement: Table S5 — Comparison of genes identified by Zaas et al with significant genes in the present manuscript. (PDF) [file pgen.1002234.s023.pdf]

Table S5

Influenza predictor genes (Zaas, 2009)	Cluster designation in this manuscript
RSAD2	3
IFI44L	3
SIGLEC1	3
LAMP3	3
IFIT1	3
IFI44	3
SERPING1	3
IFI27	3
ISG15	3
IFI44	3
HERC5	3
LOC26010	3
IFI6	3
LOC727996	**N/A***
IFIT3	3
OAS3	3
OASL	3
4-Sep	**2**
XAF1	3
OAS1	3
LY6E	3
MS4A4A	3
SIGLEC1	3
TNFAIP6	3
CCL2	**2**
OAS1	3
MX1	3
TNFAIP6	3
RTP4	3
OASL	3

* This gene cannot be mapped due to public gene annotation issue.
