# Supplementary material for: Temporal Dynamics of Host Molecular Responses Differentiate Symptomatic and Asymptomatic Influenza A Infection
Source: PLoS Genet. 2011 Aug 25;7(8):e1002234. doi: 10.1371/journal.pgen.1002234 (PMC3161909; doi:10.1371/journal.pgen.1002234)
Supplement: Table S6 — The proportions of primary white blood cell (WBC) subtypes are similar between Asx and Sx. White blood cells counts were obtained daily through standard laboratory workout. * Phenotype specific average percentage of cell subpopulation were computed using Tukey's biweight robust M-estimator. The null hypothesis H0: the frequency distribution of WBC subtypes is independent of disease phenotype was performed using Fisher-exact test. H0 is rejected at significance level of 0.01. (PDF) [file pgen.1002234.s024.pdf]

Table S6

Day	Pheno	Basophils (%)	Eosinophils (%)	Neutrophils (%)	Monocytes (%)	Lymphocytes (%)	*p*-value
-2	Asx	0.35	1.65	58.06	7.24	31.37	
	Sx	0.34	0.95	59.12	6.06	32.98	0.9697
0	Asx	0.47	2.54	54.77	7.28	32.80	
	Sx	0.52	1.46	56.48	7.52	35.73	0.8249
1	Asx	0.50	3.07	49.99	8.03	38.43	
	Sx	0.57	1.88	51.93	9.03	36.71	0.9889
2	Asx	0.61	2.69	52.47	8.60	34.97	
	Sx	0.56	0.99	65.82	8.49	21.73	0.1857
3	Asx	0.42	3.08	48.79	8.69	38.75	
	Sx	0.60	1.53	63.82	13.06	20.41	0.0221
4	Asx	0.61	3.43	49.71	8.31	37.48	
	Sx	0.65	1.75	53.30	14.95	30.36	0.5092
5	Asx	0.53	3.44	49.49	8.13	38.21	
	Sx	0.47	1.82	50.85	12.72	32.37	0.5929
6	Asx	0.62	3.37	47.66	8.95	38.92	
	Sx	0.62	1.43	44.77	11.72	41.15	0.8496
7	Asx	0.74	3.46	48.77	8.45	36.88	
	Sx	0.43	1.89	45.07	9.59	43.33	0.8088
