# Supplementary material for: Temporal Dynamics of Host Molecular Responses Differentiate Symptomatic and Asymptomatic Influenza A Infection
Source: PLoS Genet. 2011 Aug 25;7(8):e1002234. doi: 10.1371/journal.pgen.1002234 (PMC3161909; doi:10.1371/journal.pgen.1002234)
Supplement: Text S1 — Supplementary methods and supplementary discussions. (DOC) [file pgen.1002234.s025.doc]

**Text S1**

**Temporal Dynamics of Host Molecular Responses Differentiate Symptomatic and Asymptomatic Influenza A Infection**

**Yongsheng Huang^1,2^, Aimee K. Zaas^6,7^, Arvind Rao^9^, Nicolas Dobigeon^12^, Peter J. Woolf^1,4,5^, Timothy Veldman^7^, N. Christine Øien^7^, Micah T. McClain^6,7^, Jay B. Varkey^10^, Bradley Nicholson^7^, Lawrence Carin^8^, Stephen Kingsmore^11^, Christopher W. Woods^6,7^, Geoffrey S. Ginsburg^6,7^ & Alfred O. Hero III^1,2,3,4^**

^1^Center for Computational Biology and Bioinformatics, ^2^Department of Statistics, ^3^Department of Electrical Engineering and Computer Science, ^4^Department of Biomedical Engineering, ^5^Department of Chemical Engineering, University of Michigan, Ann Arbor, Michigan, USA. ^6^Institute for Genome Sciences and Policy, ^7^Department of Medicine, ^8^Department of Electrical and Computer Engineering, Duke University, Durham, North Carolina, USA. ^9^Lane Center for Computational Biology, Carnegie Mellon University, Pittsburgh, Pennsylvania, USA. ^10^Department of Medicine, School of Medicine, Emory University, Atlanta, Georgia. ^11^Center for Pediatric Genomic Medicine, Children’s Mercy Hospital, Kansas City, Missouri, USA. ^12^IRIT/INP-ENSEEIHT, University of Toulouse, Toulouse, France.

To whom correspondence should be addressed. E-mail: [hero@eecs.umich.edu](mailto:hero@eecs.umich.edu), [geoffrey.ginsburg@duke.edu](mailto:geoffrey.ginsburg@duke.edu).

**Supplementary Methods**

**Human Influenza viral challenges**

A healthy volunteer intranasal challenge with influenza A A/Wisconsin/67/2005 (H3N2) was performed at Retroscreen Virology, LTD (Brentwood, UK) in 17 pre-screened volunteers who provided informed consent. All volunteers were influenza A antibody negative at pre-inoculation testing. On day of inoculation, a dose of 10^6^ TCID_50_ Influenza A manufactured and processed under current good manufacturing practices (cGMP) by Bayer Life Sciences, Vienna, Austria) was inoculated intranasally per standard methods at a varying dose (1:10, 1:100, 1:1000, 1:10000) with four to five subjects receiving each dose. Subjects were not released from quarantine until after the 216th hour. Blood and nasal lavage collection continued throughout the duration of the quarantine. All subjects received oral oseltamivir (Roche Pharmaceuticals) 75 mg by mouth twice daily prophylaxis at day 6 following inoculation. All patients were negative by rapid antigen detection (BinaxNow Rapid Influenza Antigen; Inverness Medical Innovations, Inc) at time of discharge.

**Case definitions**

Symptoms were recorded twice daily using standardized symptom scoring [1]. The modified Jackson Score requires subjects to rank symptoms of upper respiratory infection (stuffy nose, scratchy throat, headache, cough, etc) on a scale of 0-3 of “no symptoms”, “just noticeable”, “bothersome but can still do activities” and “bothersome and cannot do daily activities”. For all cohorts, modified Jackson scores were tabulated to determine if subjects became symptomatic from the respiratory viral challenge. A modified Jackson score of >= 6 over the first five days period was the primary indicator of successful viral infection [2] and subjects with this score were denoted as “Symptomatic” (Sx). Viral titers from daily nasopharyngeal washes were used as corroborative evidence of successful infection using quantitative and quantitative PCR (Table S2) [1,2,3]. Antibody neutralization assays were also performed to corroborate this hypothesis (see discussion in the section entitled **Supplementary Discussion** below). Subjects were classified as “Asymptomatic” (Asx) if the Jackson score was less than 6 over the first five days of observation and viral shedding was not documented after the first 24 hours subsequent to inoculation. Standardized symptom scores tabulated at the end of each study to determine attack rate and time of maximal symptoms (time “T”). The clinical disease is mild (only a single fever was observed). Immune activation assays (such as antibody response) over the full time course of the challenge study were not available for our analysis.

**Biological sample collections**

During challenge study, subjects had the following samples taken 24 hours prior to inoculation with virus (baseline), immediately prior to inoculation (pre-challenge) and at set intervals following challenge: peripheral blood for serum, peripheral blood for RNA PAXgene™, nasal wash for viral culture/PCR, urine, and exhaled breath condensate. Peripheral blood was taken at baseline, then at 8 hour intervals for the initial 120 hours and then 24 hours for the remaining 2 days of the study. For all challenge cohorts, nasopharyngeal washes, urine and exhaled breath condensates were taken at baseline and every 24 hours. Samples were aliquoted and frozen at -80◦C immediately.

**RNA purification and microarray analysis**

RNA was extracted at Expression Analysis (Durham, NC) from whole blood using the PAXgene™ 96 Blood RNA Kit (PreAnalytiX, Valencia, CA) employing the manufacturer's recommended protocol. While whole blood RNA is initially extracted, a secondary procedure (B-globin reduction) was then employed to remove the contribution of red blood cell (RBC) RNA to the total RNA. A set of four peptide nucleic acid (PNA) oligomers whose sequences are complementary to the 3’ portions of the alpha and beta hemoglobin RNA transcripts were added to reduce globin RNA transcription due to RBC. The inhibition of globin cDNA synthesis dramatically reduces the relative amount of anti-sense, biotin-labeled cRNA corresponding to the hemoglobin transcripts. Hybridization and microarray data collection was performed at Expression Analysis (Durham, NC) using the GeneChip® Human Genome U133A 2.0 Array (Affymetrix, Santa Clara, CA). Raw gene expression profiles were further preprocessed using robust multi-array (RMA) analysis [4] with quantile normalization and probeset-level signals were summarized in log base 2 scale. We selected a custom Chip Definition File (CDF) version 10 for more accurate probe mapping to genome [5].

**Differential gene expression analysis between symptomatic versus asymptomatic**

Temporal gene expression was analyzed with EDGE [6] on RMA normalized intensities. A total of 5,076 genes were identified as most significantly differentially expression genes between Asx and Sx (*q*-value < 0.01). The EDGE method applies a cubic spline smoother [8] to smooth each gene temporal expression profile for each individual subject. To prevent overfitting, we fixed the number of spline knots to four such that there were at least three time points available for each knot. Subsequently, EDGE fits a group-wise cubic spline to summarize the temporal profiles of each gene over the groups of asymptomatic and symptomatic subjects, respectively. EDGE then tests the null hypothesis that there is no significant difference between Asx and Sx phenotypes by comparing the group-wise spline-fitted gene expression profiles. . In EDGE, statistical significance is assessed using an *F*-test with simultaneous multiple-testing FDR control. The final set of candidate genes (n=5,076) was selected as significantly differentially expressed between Sx and Asx with *q*-value < 0.01 [4].

The heatmaps of the 5,076 EDGE group-wise fitted trajectories are shown in Figure S18. The genes in these heatmaps are organized into groups according to the clusters found by SOM clustering, explained below. Note, the samples collected at baseline time (-24hpi) were not directly used in the EDGE differential expression analysis. Instead, they were used for quality assurance to ensure that no gene deemed significant was differentially expressed relative to pre-challenge (0hpi) samples, according to a standard paired *t*-test comparison.

**Co-clustering significant genes using Self-Organizing Map**

The self-organizing map [7] was used to cluster the complex high-dimensional temporal gene profiles of each phenotype (Figure 2). Like other metric clustering algorithms, SOM performs dimensionality reduction for visualization of complex relationships and trends by preserving the topological and metric relationships between profiles [7]. In our analysis, we aim to place, in the same region of a 2D grid layout, those genes that have similar temporal expression profiles, as measured by their Euclidean distances. As our objective is to find genes that co-cluster, i.e. cluster over both Asx and Sx phenotypes, we applied the SOM clustering procedure to the feature vector defined as the concatenation of the two Asx and Sx time trajectories. These trajectories were obtained by sampling the cubic-spline fits generated by the EDGE procedure described above. Since it is the shape of the temporal trajectories that is of interest, prior to clustering the fitted trajectories were subsequently *z*-score normalized over time, i.e. for each gene and subject the time average value was subtracted and the result was normalized by the root-mean-square temporal variation.. As there exists no gold standard in choosing the “best” map configuration among all possible maps, we proposed an analytical selection procedure in which we balance the complexity of the map (number of prototypes), the distances between genes and their prototypes, and the silhouette values [8] of genes (measure of the closeness of a gene to its within-cluster neighbors and to its neighbor-cluster). This resulted in a 4x2 hexagonal grid of prototypes or clusters. Each prototype’s representative centroid was initially chosen from genes at random. The initial neighborhood size was set such that each neighborhood contained 25% of prototypes. The total number of iterations was chosen such that each gene was repeatedly presented to the map 50 times. Each gene was clustered into a prototype to which it is closest in Euclidean (or *L*2) distance measurement. The average expression values of each individual cluster, along with corresponding ± two standard deviations intervals, were plotted in Figure 2C. We also estimated the centroids of each SOM cluster and corresponding 95% confidence intervals using a nonparametric bootstrap method without assuming normality [9]. The bootstrap derived centroids of the clusters are almost identical and the confidence limits of these centroids are much smaller than and completely covered by the ± two standard deviations shown in Figure 2C. Figure S18 shows a more granular view of these clusters, rendering the heatmaps of the EDGE group-wise cubic-spline fits of all 5,076 significant genes, organized according to SOM cluster. Within each cluster, the genes shown in the heatmaps are arranged from top to bottom of the Figure in increasing order of EDGE *p*-value (decreasing order of significance).

**Polar plot visualization of temporal expression pattern of a cluster**

The polar plots (Figure 2A) provide a different visualization of the differences between temporal gene expression profiles for Asx and Sx phenotypes. Each polar plot depicts the expression pattern shared by genes of a SOM cluster. Within a plot, the temporal expression of Asx resides on the top portion of the circle while Sx expression occupies the bottom half. Each phenotype’s expression values are placed in time sequence, increasing in the counterclock-wise direction, inside its own half circle. Consequently, the expression profiles of Asx and Sx at any given time point can be compared at opposite ends of a radial line passing through the polar origin. Such symmetric arrangement facilitates visual examination of contrasts in phenotypic gene expression patterns. We emphasize that it is not adequate to only look at one phenotype alone or the ratio of Sx/Asx expression values. This is because of the fact that both Asx and Sx undergo significant changes in gene expression profiles, a consequence of universal protective immune response. In Figure 2B we show heatmaps for the top 5 genes from each SOM cluster having the most significant differential expression. The expression values from different time points are aligned horizontally. Although this type of visualization arrangement is in line with traditional clustering results, we can see that it is less convenient to contrast the expression values of two phenotypes at any given time point as it requires visual search through the horizontal time line. The reader may find that segment plots of SOM clusters add interpretability to the heatmaps of temporal expression patterns, allowing more direct simultaneous comparisons between particular time points and phenotypes. **Biological pathway enrichment analysis**

To identify biological pathways that are enriched in each individual clusters, we used Ingenuity© Pathway Analysis (IPA) tool and queried their proprietary knowledge database of functional interactions between molecules. The representative pathways were shown in Table 1.

**Testing monotonicity of expression pattern of clusters**

We used the non-parametric Jonckheere-Terpstra (JT) method (R package SAGx by Per Broberg) [10,11] to quantitatively test whether a monotonic increase or decrease trend exists in a cluster expression centroid. For each of the eight clusters, the JT test was carried out to test the null hypothesis that there exists no monotonic trend in changes of gene expression over time. The alternative hypotheses are that the median gene expression of later time points was higher or lower than that of earlier time points:

$$M_{t}^{i}<M_{t+1}^{i}<\ldots<M_{t+n}^{i} or M_{t}^{i}>M_{t+1}^{i}>\ldots>M_{t+n}^{i}$$

where$M_{t}^{i}$is the centroid of gene expression of cluster $i$at time$t$.

This test was performed for each one of two phenotypes separately with significance measures (*p*-values) shown in Table S3. The *p*-values were further adjusted with Benjamini-Hochberg method [12] to correct for multiple hypothesis testing.

We note that JT test is different from EDGE test in that JT tests any monotonicity trend in temporal gene expression subject to the constraint that the change in expression has to be either monotonically increasing or monotonically decreasing. On the other hand, EDGE tests any phenotypic difference in expression over all time points. Therefore EDGE has more power in detecting differential expression over time without requiring monotonicity whereas JT has more power in detecting monotonic temporal expression within each Asx and Sx phenotype without requiring differential expression.

**Associating disease phenotypes with canonical biological pathway**

To identify the canonical gene pathways in each SOM cluster that are highly associated with disease phenotypes, we applied Globaltest [13] using all pathways included in MsigDB database (v2.5) [14]. Briefly, all significant genes from a SOM cluster were first mapped onto individual MsigDB pathways. Then we carried out the group testing procedure outlined in [13] to test the association between each pathway and phenotypes using logistic regression via hierarchical generalized linear model fitting. Specifically, the model is formulated as

$$H_{0}:\beta_{1}=\beta_{2}=\ldots=\beta_{m}=0$$

where$Y_{i}\in(0,1)$ for the Asx and Sx phenotypes, respectively, and $x_{ij}$is the expression value for $j$-th gene of $i$-th subject and $j\in C$for a predetermined pathway $C$.

The null hypothesis is simply that all $\beta$’s equal to zero, corresponding to no correlation between the pathway $C$ (includes all$j$genes) and disease phenotypes. The significance of association was assessed using permutation test and we further adjusted the p-values to account for multiple testing according to the Benjamini-Hochberg procedure [12]. This analysis was conducted for each SOM cluster individually and for all 16 time points in the challenge study where the gene expression profiles were examined.

**Correlating disease symptom scores with temporal expression values of clusters**

We estimated the correlation between clinically determined symptom scores and the temporal gene expression of SOM clusters using a standard linear mixed model. Specifically, for each one of the 10 categories of symptom scores, we regressed the scores onto the expression value vector of every one of eight SOM clusters, separately, with a random-effects term accounting for within-subject temporal correlation. For each symptom and cluster prototype the model is

where

- $y_{i}^{(t)}$ is a $t\times1$vector of measures on a symptom category for subject over time points
- $x_{i}^{(t)}$is a $t\times1$vector of average gene expression of a cluster for subject over time points
- $\beta$is a scalar coefficient of fixed-effects of expression values
- $b_{i}$is a scalar coefficient of random-effects for subject

The goodness-of-fit of the mixed model was assessed using the *signed coefficient of determination* [15,16] defined as $r_{i}=\text{sign}\left(\hat{\beta}\right) \bullet\sqrt{\frac{\sum^{ } \left(\widehat{y_{i}}-\bar{y}\right)^{2}}{\sum^{ } \left(y_{i}-\bar{y}\right)^{2}}}$

where

- $r_{i}$ is a correlation coefficient taking values between minus and plus one.
-
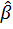
 is the estimated fixed-effects coefficient in the fitted model (see above).
- $\sum^{ } \left(\widehat{y_{i}}-\bar{y}\right)^{2}/\sum^{ } \left(y_{i}-\bar{y}\right)^{2}$is the *unsigned coefficient of determination*, which is equivalent to the squared correlation between the response variable and the predictor in the fitted mixed model regression.

The quantity $r_{i}$ is an approximation to the correlation coefficient between symptom scores and cluster expression values.

**Unsupervised detection of disease signature with Bayesian Linear Unmixing (BLU)**

All the above analysis methods involved the use of clinical labels, namely Sx and Asx. BLU extends and validates the SOM results using an unsupervised learning technique that does not involve these labels. In particular, without the benefit of ground truth clinical symptom scores, BLU discovered many of the same genes as SOM and specified a gene expression factor that separated asymptomatic from symptomatic subjects. Within the symptomatic group, the genes that principally contributed to this factor exhibited temporal expression patterns correlated highly with symptom patterns and differentiate the time samples between pre- and post-onset time of the inflammatory response.

Originally developed for unmixing composite spectra in hyperspectral imaging [17], the BLU is a Bayesian factor analysis method. Like other unsupervised Bayesian factor analysis methods, BLU finds a decomposition of the data matrix **Y**, here a ***p*** by ***n*** matrix of abundances of the ***p*** mRNA transcripts for each of ***n*** gene expression profiles, into a matrix product **MA** where each column of **M** is a factor and each column of **A** is a set of factor loadings corresponding to individual factors in **M** for a given chip:

$$Y=MA+N$$

In essence, BLU estimates two matrix valued latent variables **M** and **A,** whose product best approximates the most important information contained in the observation **Y** while minimizing the residual model fitting error (denoted as **N** in the formula above) with latent variable order selection according to an hierarchical Bayesian model. However, unlike other factor analysis methods, BLU decomposes the data into relative proportions, the columns of **M** and the columns of **A** are non-negative and the columns of **A** sum to one. More specifically, each BLU-discovered factor can be viewed as a gene expression profile, whose amplitudes represent the relative contribution of each gene present in that factor, and the factor loadings are the proportions of these factors that are present in each chip. Such positivity constraints are natural in gene microarray analysis as the expression intensity measurements of genes are always non-negative.

BLU was run on all genes on the expression array and extracted a total of three major BLU factors. The factor scores of the samples were subsequently divided into two groups: samples taken before inoculation (pre-inoculation samples) and samples taken after inoculation (post-inoculation samples). We then tested for significant difference between the scores of the pre-inoculation and post-inoculation samples (*t*-test with *p*-value less than 0.01). At this significance level only one of the factors passed this test – the acute respiratory factor shown in Figure 1A. Based on the score of this acute respiratory factor, we quantitatively determine the four regions by the following thresholding algorithm:

-----------------Begin algorithm----------

**Step 1**: Identify Region 1 (specified as pre-inoculation times). Compute the mean and standard deviation of scores on all samples in Region 1, denote these quantities $\mu_{1}$and .

**Step 2**: Identify Region 2 and Region (3 union 4). Classify a subject as having an acute viral signature if he/she has at least one post-inoculation time sample whose factor score *f* exceeds $\mu_{1}$by at least $\sqrt{20} s_{1}$

$$f\geq\mu_{1}+\sqrt{20} s_{1}$$

Those samples that exceed this threshold are statistically different from the pre-inoculation samples with *p*-value at most 0.05 (by Tchebychev's inequality). This determines Region 2 (post-inoculation times of subjects with no acute signature) and region (3 union 4) (post-inoculation times of subjects with acute signature).

**Step 3**: Identify the boundary between Region 3 and Region 4. The boundary between Region 3 (pre-onset but post-inoculation times) and Region 4 (post-onset), called the acute response onset time, is defined as the first post-inoculation time at which the factor score exceeds the threshold specified in Step 2.

--------------------End Algorithm-------------------

Each row of Figure 1A shows the factor score associated with each gene expression chip with respect to the principal factor. The chips with the smallest scores can be found in asymptomatic and pre-symptom regions, 2 and 3 respectively, while those having maximum scores close to 1 are in post-symptom region 4 (Figure 1A). Strikingly, the subjects corresponding to those chips with high factor scores are exactly those who were later confirmed to have developed clinical overt symptoms. Of note, the boundary between regions 3 and 4 (Figure 1A) of the Sx subjects is the abrupt symptom onset time detected by this principal BLU factor. Furthermore, a list of most dominant genes that are enriched in this factor was determined by using a simple dominance ranking on the factor loading of each gene over the three factors. Specifically, a gene is said to enrich the principal factor if its loading in this factor is the highest among the three factors.

Based on results of BLU, we defined 4 classes: Class 1 (pre-inoculation) corresponds to chips acquired from subjects before the inoculation time; Class 2 (post-inoculation Asx) corresponds to chips from asymptomatic subjects acquired after-inoculation; Class 3 (post-inoculation and pre-symptom) corresponds to chips from symptomatic subjects acquired after inoculation but before symptoms occur; and Class 4 (post-symptom) corresponds to chips from symptomatic subjects after symptoms occur (Figure 1A).

**Extracting largest contrast genes for each class pair using LogitBoost**

Based on the 4-class designation defined by BLU, we conducted a gene importance study and identified genes exhibiting largest contrast between each pair of classes using the *LogitBoost* method [18,19,20] as a contrast function. As it uses boosting algorithm to perform variable selection, our implementation of LogitBoost yields a set of genes in addition to a classifier function. The objective of using this model was two-fold: 1) to determine the principal genes that are most associated with these classes; and 2) to quantify the variability in such associations between the genes and the classes. We emphasize that the implementation of our pairwise boosting model is for exploration of variable importance and identification of largest contrast genes – i.e., we are not proposing it as a prediction model or classifier of global disease state.

A logistic model was constructed for each class pair using a functional gradient descent algorithm [18,19,20]. Briefly, the fitting procedure takes multiple boosting iterations. At each iteration, one gene is selected on the basis that it best differentiates chips between two classes. This gene is used to construct a base learner for the final model. Such component-wise univariate addition of gene provided an effective variable selection mechanism that supplements the model with the next best predictor gene at each step. The final estimate takes an additive form of all base learners fitted at each iteration [18,19,20]. Furthermore, we generated 200 bootstrap samples from each class [9]. We randomly selected 2/3 of each bootstrap sample to construct the boosting ensemble and the other 2/3 of data was used to evaluate the variability of the association between the largest contrast genes and each class pair. We defined the largest contrast genes as the set of genes that were selected by LogitBoost algorithm for each class pair more than 100 (50%) of the 200 bootstrap samples. Heatmaps of the average expression of these high contrast genes are shown in Figure 1C. These heatmaps display gene expression trajectories, averaged over phenotype and smoothed over time, using the same method and cubic spline smoothing parameters as were used to render the EDGE gene heatmaps shown in Figures 2B and S18 (See above section entitled “**Differential gene expression analysis between symptomatic versus asymptomatic**” for details).

**Supplementary Discussion**

**Comparison of this study with the study reported by Zaas et al. [21].** This work probes the temporal nature of the host genomic response as compared to Zaas et al that looked at a single time point (peak infection).  The question we are addressing here is whether and how the data evolve over time and whether the asymptomatic state represents a passive or active response to pathogens.  Our data not only show that the peak Sx response from Zaas appears to be manifest as an evolving signature of two gene clusters (2 and 3 which are predominantly inflammatory response), but additionally that mechanisms characterized by clusters 1 and 4-8 are temporally activated as well.  Our data further shows that there is an active temporal response in Asx that differs from the Sx response and is particularly strong in clusters 2, 6, and 8.

Zaas et al found 30 biomarkers that best discriminated between symptomatic and asymptomatic individuals at peak symptom time. The vast majority (29 out of 30) of these biomarkers are found in clusters 3 ($A^{nc},S_{mid}^{up}$) and cluster 2 ($A_{early}^{dw},S_{mid}^{up}$)of our paper (Table S5). Not surprisingly, these two clusters are those that show the largest contrast between Asx and Sx expression levels near peak symptom time (Figure 2). Other clusters reported in our paper correspond to genes that respond differently in Asx and Sx at earlier time points. Thus, while being completely consistent with the results from Zaas et al [21], our analysis goes beyond peak symptom time and establish striking temporal differences in host response programs between Asx and Sx subjects.

In Zaas et al, the performance of the peak time Sx vs Asx classifier was validated on an independent dataset presented in Ramilo et al [22]. As 29 of the 30 genes identified in Zaas et al are in our clusters this also validates our results at peak symptom time. Unfortunately, as the Ramilo dataset only consists of clinical samples taken near peak symptom time it cannot be used to validate our temporal analysis at other time points.

**The nature of asymptomatic phenotype** We performed several tests to rule out the possibility that our results may merely reflect failed inoculation in the asymptomatic subjects instead of innate differences in host response. Although it is difficult to rule this out with 100% certainty, our data suggests that it is highly unlikely that the inoculation failed to establish productive infection in Asx hosts. The evidence against failed inoculation is a follows.

1. The temporal gene expression analysis presented in the main text has shown that Asx transcription state is not passive. Instead, it actively evolves in response to viral challenge. As presented in the manuscript, the viral inoculation elicited a strong molecular host response in the Asx subjects. When the expression profiles from asymptomatic subjects were studied alone, a total of more than 3,000 genes showed statistically significant post-infection expression changes. In particular, such expression change does not correlate with viral detection. For example, two subjects **#3** and **#17** never yield detectable virus (<1.25) in their nasal wash (Table S2). However, the Asx-specific temporal suppression of gene NLRP3, a key factor involved in activating *inflammasome* protein complex, is among the most significant for these two subjects (Figure S14).

Moreover, the gene expression responses of the two seroconverted Asx subjects (#2 and #3), according to haemagglutination inhibition (HAI) assay, are not significantly different from those of other asymptomatic individuals (Figure S14). As additional evidence, Figure S15 shows individual Asx subjects’ temporal expression of RPL3. The overall average of the Asx profiles is temporally changing at FDR level of significance (*q*-value) 0.0002 and again the subjects #2 and #3 do not appear to have atypical trajectories. Considering the fact that these subjects tested negative for binding antibody (Ab) to HA prior to inoculation, this indicates that the non-passive transcriptional responses we observed in Asx hosts are not directly related to their serum binding Ab activity. We also found no significant correlation between serological conversion and the final disease outcome (*p*-value = 0.27), suggesting that host gene expression signature serves as a better marker for symptomatic infection than serology measures do.

2. In the paper we presented a set of predictor variables (Table S4 first column - labeled 1v2) that differentiate between pre-inoculation baseline samples and asymptomatic post-inoculation samples. Our study of gene importance showed high level of discrimination performance of these genes, suggesting that a robust immune response program was indeed activated in the Asx subjects. A few of these predictor variables, e.g. GM2A, IRS2, and FOXO3, have been previously implicated in innate immunity and insulin receptor signaling. We think that these variables represent potential new targets for studying viral control mechanisms in Asx host response.

3. The viral shedding rates observed in our study are not inconsistent with that of previous studies. Specifically, 50% (4 out of 8) of the Asx subjects had evident viral shedding and this is on par with that of “subclinical” or “secondary” infections reported by Lau et al [23]. The level of shedding is has been referred to in the literature as “asymptomatic infection”. Also, 75% (6 out of 8) of the Asx subjects reported some symptoms during the study. This provides further support for our clinical determination of the Asx subjects as “asymptomatic”.

4. We can also rule out any possible dosage effect as the inoculation dosage was found to be un-related to the infection outcome (Figure S13). Subjects who received relatively lower amount of inoculation do not necessarily become more ill than the ones who received higher dose of virus and there is no statistically significant dependence between disease outcome and inoculation dosage. The test for dosage effect failed according to two standard statistical test of significance: Fisher’s exact test (resulting in rejection of the correlation hypothesis at any level less than the *p*-value of 0.2299); and very low R value ($R^{2}=-0.0662$) of a linear regression of the disease outcome on dosage level.

5. Assay of the serum neutralizing antibody (nAb) titre was performed on all samples at early time points and many subjects showed relatively high level of nAb (≥ 40) at the time of challenge. However, there is no significant difference between the nAb titers found in Asx or Sx subjects (Figure S12A,B). Because these subjects were recruited from a natural population and thus had likely prior exposure to viral pathogens such level of nAb activity is not surprising.

More concretely, a Wilcoxon rank test generated a p-value of 0.80 at day 0 and 0.82 at day 7 on the hypothesis that there is no difference between nAb levels in the Asx and Sx groups. Furthermore, the pre-inoculation nAb does not have significant effect (R squared of linear regression line in black equal to 0.06) on disease symptom severity as measured by the clinical Jackson scores (Figure S12C). Most importantly, the nAb titer is observed to increase over time in both Asx and Sx individuals (Figure S12D). At minimum, this indicates a boosting effect of immunity, and suggests that even if viral replication was inhibited, enough viruses were detected by the Asx host immune system to cause expansion of Ab producing cells.

6. The reported attack rate in our study is consistent with other similar studies reported in the literature, e.g., Turner et al [24] and Carrat et al [25]. On the basis of an extensive survey of 56 human influenza challenge studies with 1,280 health volunteers, Carrat et al reported that the frequency of symptomatic infection was 66.9% (95% confidence interval: 58.3, 74.5).

Taken together, we think that this provides strong evidence that the inoculation did elicit a unique and robust host molecular response in Asx subjects. This response is significantly different from that of Sx individuals. The fact that some Asx subjects were not infected does not render this group of subjects any less interesting than their symptomatic counterparts. We believe that there exist important biological and immunological reasons that some volunteers can withstand considerable amount of viral insult and show no severe disease symptoms and we hope that the findings will lead to additional studies that clarify the apparent immunity of asymptomatic responders.

**Viral shedding analysis.** The measured viral shedding patterns shown in Figure S13 are highly variable. We did not find statistically significant correlations between viral shedding patterns and either inoculation dosage or molecular signature patterns. We did observe that all symptomatic subjects and half of the asymptomatic subjects shed virus at the site of infection (nose). Peak levels of shedding in the symptomatic subjects were significantly higher than those of the asymptomatic subjects. The shedding of virus by asymptomatic carriers is a fairly well-known phenomenon both in naturally occurring disease [23] as well as in influenza challenge trials [25]. In symptomatic individuals, the average shedding profile peaks soon after inoculation and decays steadily afterwards. This is consistent with the meta analysis of Carrat et al. [25]. Indeed, viral replication in respiratory infection is known to occur soon after exposure at the site of infection; in the nose in our study. The innate immune system is initially overwhelmed (and in some cases purposefully modified) by the virus allowing initial replication. Signaling/Recruitment of additional inflammatory cells, initially nonspecific and then adaptive immune cells, quickly limit viral replication at the local site of infection. It is this initial viral replication and stimulation of the innate immune response that then triggers the signaling cascades and subsequent stimulation of cells in the periphery, i.e., in blood where the RNA of this study was collected. Hence, it is not surprising for the observed peripheral signature to lag behind biological events at the site of initial infection. This is doubly true when the part of the signal we are measuring in the signature deals with stimulation and development of adaptive immunity in response to viral exposure, which is known to occur in a delayed fashion.

**References**

1. Jackson GG, Dowling HF, Spiesman IG, Boand AV (1958) Transmission of the common cold to volunteers under controlled conditions. I. The common cold as a clinical entity. AMA Arch Intern Med 101: 267-278.

2. Turner RB (2001) Ineffectiveness of intranasal zinc gluconate for prevention of experimental rhinovirus colds. Clin Infect Dis 33: 1865-1870.

3. Barrett B, Brown R, Voland R, Maberry R, Turner R (2006) Relations among questionnaire and laboratory measures of rhinovirus infection. Eur Respir J 28: 358-363.

4. Bolstad BM, Irizarry RA, Astrand M, Speed TP (2003) A comparison of normalization methods for high density oligonucleotide array data based on variance and bias. Bioinformatics 19: 185-193.

5. Dai M, Wang P, Boyd AD, Kostov G, Athey B, et al. (2005) Evolving gene/transcript definitions significantly alter the interpretation of GeneChip data. Nucleic Acids Res 33: e175.

6. Storey JD, Xiao W, Leek JT, Tompkins RG, Davis RW (2005) Significance analysis of time course microarray experiments. Proc Natl Acad Sci U S A 102: 12837-12842.

7. Kohonen T (1995) Self-Organizing Maps. Series in Information Sciences 30.

8. Rousseeuw P (1987) Silhouettes: a graphical aid to the interpretation and validation of cluster analysis. Journal of Computational and Applied Mathematics 20: 53-65.

9. Efron B (1979) Bootstrap Methods: Another Look at the Jackknife. The Annals of Statistics 7: 1-26.

10. Lehmann E (1975) Nonparametrics: Statistical Methods Based on Ranks. Holden Day: 233.

11. Hero A, Fleury G (2004) Pareto-optimal methods for gene ranking. Journal of VLSI Signal Processing 38: 259-275.

12. Benjamini Y, Hochberg Y (1995) Controlling the false discovery rate: a practical and powerful approach to multiple testing. . Journal of the Royal Statistical Society Series B 57: 289–300.

13. Goeman JJ, van de Geer SA, de Kort F, van Houwelingen HC (2004) A global test for groups of genes: testing association with a clinical outcome. Bioinformatics 20: 93-99.

14. Subramanian A, Tamayo P, Mootha VK, Mukherjee S, Ebert BL, et al. (2005) Gene set enrichment analysis: a knowledge-based approach for interpreting genome-wide expression profiles. Proc Natl Acad Sci U S A 102: 15545-15550.

15. Faraway J (2004) Linear Models with R. Chapman and Hall.

16. Hössjer O (2008) On the coefficient of determination for mixed regression models. Journal of Statistical Planning and Inference 138: 3022-3038.

17. Dobigeon N, Moussaoui S, Coulon M, Tourneret J-Y, Hero A (2009) Joint Bayesian endmember extraction and linear unmixing for hyperspectral imagery. IEEE Trans on Signal Processing 57: 4355-4368.

18. Bühlmann P (2006) Boosting for high-dimensional linear models. Annals of Statistics 34: 559-583.

19. Bühlmann P, Yu B (2003) Boosting with the L2 loss: regression and classification. Journal of the American Statistical Association 98: 324–339.

20. Bühlmann P, Hothorn T (2007) Boosting algorithms: regularization, prediction and model fitting. . Statistical Science 22: 477–505.

21. Zaas AK, Chen M, Varkey J, Veldman T, Hero AO, 3rd, et al. (2009) Gene expression signatures diagnose influenza and other symptomatic respiratory viral infections in humans. Cell Host Microbe 6: 207-217.

22. Ramilo O, Allman W, Chung W, Mejias A, Ardura M, et al. (2007) Gene expression patterns in blood leukocytes discriminate patients with acute infections. Blood 109: 2066-2077.

23. Lau LL, Cowling BJ, Fang VJ, Chan KH, Lau EH, et al. Viral shedding and clinical illness in naturally acquired influenza virus infections. J Infect Dis 201: 1509-1516.

24. Turner RB, Bauer R, Woelkart K, Hulsey TC, Gangemi JD (2005) An evaluation of Echinacea angustifolia in experimental rhinovirus infections. N Engl J Med 353: 341-348.

25. Carrat F, Vergu E, Ferguson NM, Lemaitre M, Cauchemez S, et al. (2008) Time lines of infection and disease in human influenza: a review of volunteer challenge studies. Am J Epidemiol 167: 775-785.
